# Acute and chronic effects of high-intensity interval training on selected exerkine secretion in health, disease, and aging: a systematic review

**DOI:** 10.3389/fphys.2025.1733269

**Published:** 2026-01-23

**Authors:** Zbigniew Jost, Agata Rozynkowska, Michalina Głąb, Alicja Sitkiewicz, Mia Goiko, Radosław Laskowski, Fabian Herold, Zsolt Radák, Sylwester Kujach

**Affiliations:** 1 Department of Biochemistry, Gdansk University of Physical Education and Sport, Gdansk, Poland; 2 Faculty of Physical Culture, Gdansk University of Physical Education and Sport, Gdansk, Poland; 3 Department of Physiology, Gdansk University of Physical Education and Sport, Gdansk, Poland; 4 Department of Physiology, Faculty of Medicine, HMU Health and Medical University Erfurt, Erfurt, Thuringia, Germany; 5 Research Institute of Sport Science, Hungarian University of Sport Science, Budapest, Hungary; 6 Faculty of Sport Sciences, Waseda University, Tokorozawa, Japan; 7 Department of Physiology, Medical University of Gdansk, Gdansk, Poland

**Keywords:** aging, disease, exerkine, health, high-intensity interval training

## Abstract

**Introduction:**

In contemporary research practice, high-intensity interval training (HIIT) has received growing attention compared to other types of endurance training [e.g., moderate-intensity continuous training (MICT)]. This is primarily related to HIIT’s ability to induce higher metabolic stress, driving an increased exerkine secretory response (i.e., of specific proteins) compared to MICT. To date, previous reviews on HIIT have primarily focused on single exerkines, while a more comprehensive analysis, as required to gain a more comprehensive understanding of the complex exercise-related physiological processes, is absent.

**Methods:**

To reduce non-exercise protocol-related outcome heterogeneity, the rigorous inclusion criteria (i.e., exercise intensity in the HIIT adjusted for the target population of healthy, diseased, or older individuals, and not taking any medications) were applied.

**Results:**

A total of 39 studies were selected for the systematic review, with fourteen, twenty-two, and three for the acute, chronic, and both acute and chronic effects of HIIT on exerkine concentrations, respectively. Acute HIIT appears to result in greater changes in BDNF and VEGF concentration than the control group performing lower-intensity exercise or no exercise. Metabolically active exerkine, such as adiponectin, mainly fluctuates among overweight and obese participants.

**Discussion:**

This systematic review did not yield any definitive results regarding alterations in IGF-1, irisin, cortisol, and interleukin levels. Tendentially, HIIT is more effective than MICT and non-exercise interventions to induce a greater secretory response of certain exerkines, such as BDNF, VEGF and adiponectin. Evidence regarding exerkine secretion in response to HIIT among older adults remains limited, highlighting the need for further investigation.

**Systematic Review Registration:**

Identifier CRD420251003743.

## 1 Introduction

### 1.1 The history of MICT and HIIT

Following the revival of the Olympic Games, different stakeholders, such as athletes, coaches, and scientists, aimed at identifying an effective training approaches to improve the body’s capacities and, in turn, athletic performance. In this context, the moderate-intensity continuous training (MICT) method, which emerged at the turn of the 20th century, became a key training method for Olympic preparation, particularly for long-distance running.

**FIGURE 1:**
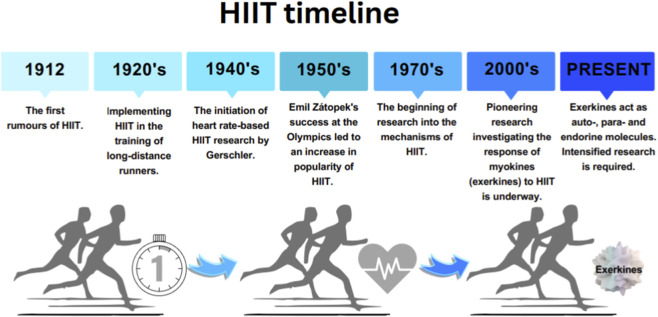
Timeline of the HIIT.

This form of training was employed without the precisely defined intensity guidelines known today. In his book ‘*The Book of Athletics’*, Paul Withington describes how he introduced the concept of MICT, tailoring adjustments to training volume for young athletes. He placed particular emphasis on optimising sessions so that, with each subsequent effort, the distance covered, and the intensity would increase (Withington, 1922). Nevertheless, as the intensity of training increased, it became more challenging to maintain the appropriate volume of each training session. Consequently, alternative methods to MICT were explored, such as dividing sessions into intervals, otherwise known as interval training. The concept of intervals, particularly in the context of high-intensity interval training (HIIT), was primarily popularised by coach Lauri Pikhala and the success of Finnish long-distance runners in the 1920s and 1930s. However, the first rumours about HIIT date back to the early 20th century (Laursen and Buchheit, 2019) (Figure 1).

Initially, interval training programmes were developed through trial and error to uncover their potential benefits. In the 1930s, for example, cardiologist Hans Reindell and coach professor Woldemar Gerschler applied HIIT systematically to both patients and athletes (Reindell and Roskamm, 1962). These sessions were based directly on monitoring participants’ heart rates (HRs), with the aim of reaching a peak of 180 beats per minute (bpm). This was followed by a recovery period during which the HR decreased to ∼120 bpm (Gibala and Hawley, 2017), with such work-rest cycles were typically repeated multiple times. In general, various interval training protocols have been investigated, with HIIT and sprint interval training (SIT) being the most extensively studied one (Coates et al., 2023; Hall et al., 2023). HIIT typically involves ‘near-maximal’ efforts performed at 80%–95% of maximal heart rate (HR_max_), whereas SIT consists of ‘all-out’ or supramaximal bouts carried out at intensities sufficient to elicit maximal or peak oxygen uptake (VO_2max/peak_). The components characterized an interval training include but are not limited to the type (modality), intensity, number of work bouts per exercise sessions, the type (i.e., active or passive) and duration of the recovery/rest bouts [i.e., acute density; (Herold et al., 2025)] in a session, the frequency, density and overall duration of the intervention (Herold et al., 2025; Buchheit and Laursen, 2013a; Buchheit and Laursen, 2013b), and application of training principles and periodization strategies (Buchheit and Laursen, 2013b; Herold et al., 2019). The careful adjustment of those exercise and training variables allows HIIT protocols to be tailored to participants’ abilities and needs; however, it also introduces challenges when evaluating their effectiveness (Billat, 2001; Viana et al., 2018).

Therefore, investigating whether engaging in HIIT provides a higher efficacy than engaging in MICT, particularly with regard to metabolic changes, can be challenging may explain why this topic remains under-researched. Thus, this review aims to address this research gap.

### 1.2 Metabolic mediators of HIIT response

The response of the skeletal muscle, the cardiovascular system, the brain, and other human tissues to acute and chronic exercise is regulated by the secretion of specific proteins. These proteins are also referred to as ‘*exerkines*’, if they are released in response to physical exercise (Chow et al., 2022), which is a planned, and structured form of physical activity (Caspersen et al., 1985). One of the exerkines that has been extensively studied in contemporary research practice is the brain-derived neurotrophic factor (BDNF), which was first isolated from a pig’s brain in 1982 (Barde et al., 1982). Since then, BDNF has been the subject of extensive research efforts, particularly concerning the site of its secretion, with the brain has been identified as the largest cluster of nerve cells being responsible for the majority of BDNF secretion (Pedersen, 2011). Furthermore, decades of research have shown that BDNF, which has auto-, para-, and endocrine effects, can also be secreted by peripheral tissues such as skeletal muscles and platelets (Fujimura et al., 2002; Matthews et al., 2009; Cho et al., 2012).

BDNF influences memory by inducing changes in membrane receptor expression and translocation, as well as activating several pathways that act synergistically to facilitate cellular outcomes affecting synaptic plasticity (Loprinzi and Frith, 2019). In pathological states such as neurological disorders, reduced levels of BDNF can be increased through engagement in regular physical activity, a proper diet, and certain medications (Gao et al., 2022).

Furthermore, longitudinal studies on aging have found that the natural process of aging itself, unencumbered by disease, also triggers a decline in serum BDNF levels (Erickson et al., 2010). Higher baseline plasma BDNF levels have also been associated with less decline in brain volume (Zhang et al., 2023). The decline in BDNF levels with age may be related to natural aging processes in the brain, reduced physical activity, and diminished cognitive function in older adults. These findings suggest that maintaining a healthy lifestyle including relatively high levels of physical activity can potentially delay the decline in BDNF levels in older adults. However, it is unrealistic to assess health based on a single exerkine, so a multifaceted approach considering several exerkines, such as vascular endothelial growth factor, is necessary for a better understanding of the modulating effect of chronic exercise, such as HIIT, on health and aging or disease-related processes influencing the latter.

Vascular endothelial growth factor (VEGF), first described in 1989 (Ferrara and Henzel, 1989), is a protein produced by various cell types that plays a key role in stimulating angiogenesis and increasing vascular permeability (Ferrara et al., 2003). Some studies suggest that VEGF is an essential growth factor for neurons and glial cells, protecting hippocampal neurons from hypoxic damage *via* an anti-excitotoxic mechanism (Svensson et al., 2002). Furthermore, Dvorak et al. emphasised the significant role of VEGF in regulating physiological and pathological angiogenesis in cancer and inflammatory processes (Dvorak et al., 1999), establishing its potential as an anti-disease and anti-aging agent. Reduced levels of VEGF have been linked to motor neuron degeneration, as they decrease neural tissue perfusion and VEGF-dependent neuroprotection (Storkebaum et al., 2004). Moreover, VEGF levels and the activity of its signalling pathways decrease with age; however, increased VEGF signalling can prevent age-associated capillary loss (Grunewald et al., 2021). Hypoxia is one of the key drivers of the mRNA production required for VEGF in both normal and pathological conditions (Karar and Maity, 2011). Therefore, physical training, as a planned, structured, and purposeful form of chronic physical exercise (Budde et al., 2016), often involving exercises with a vigorous intensity, such as HIIT, is a natural intervention to promote transient and tissue-specific hypoxia, triggering a higher release of other factors (e.g., VEGF) (Norrbom et al., 2022).

In addition to BDNF and VEGF, other exerkines are known to exhibit both pro- and anti-inflammatory properties, playing a crucial role in maintaining tissue homeostasis. Among them, cytokines as a specific exerkine type, particularly interleukins and metabolically active proteins such as adiponectin and leptin, are key regulators of systemic energy balance, immune function, and metabolic health (Liu and Li, 2025). Alterations in their levels or interactions may indicate an increased risk of metabolic disorders, including type II diabetes (Zhou et al., 2024), and are also closely linked to the accelerated aging (Gulcelik et al., 2013). Thus, maintaining an optimal balance between these molecules appears essential for preventing chronic low-grade inflammation and preserving metabolic flexibility, especially in older adults. Although some studies have investigated interventions aiming to restore this balance, particularly in aging populations and individuals with metabolic or inflammatory diseases (Cipryan et al., 2021; Senkus et al., 2022), evidence regarding the specific impact of HIIT on these mechanisms remains limited and warrants further research.

To date, several systematic reviews have been published (Fernández-Rodríguez et al., 2022; Mielniczek and Aune, 2024; Rodríguez-Gutiérrez et al., 2024), carefully analysing the effects of HIIT on the role of mediators of health effects, such as the exerkine BDNF, in healthy, older, or diseased populations. However, as far as we know, none of the previous systematic reviews have considered medication, an important modulator of specific exerkine response, as an exclusion criterion to reduce non-exercise protocol-related outcome heterogeneity, even among diseased or older adults. Therefore, this review aimed to systematically summarise current knowledge on health-promoting selected exerkines involved in neuroprotection and metabolic health, while considering the moderating role of exercise intensity and medication. Also, to provide a comprehensive perspective on protein secretion, we included both the acute and chronic responses to HIIT and MICT. Our aim was also to investigate whether exerkine secretion responses differ between HIIT and other exercise protocols and controls in healthy, older, and diseased populations.

## 2 Methods

The protocol for this systematic review was registered at the International Prospective Register of Systematic Reviews (PROSPERO; registration number: CRD420251003743) and follows the recommendations of the Preferred Reporting Items for Systematic Reviews and Meta-Analyses (PRISMA) statement (Page et al., 2021) (Supplementary Table S1).

### 2.1 Search strategy and data collection process

Relevant literature was identified in four databases: Medline (*via* PubMed), Web of Science, Google Scholar, and Scopus from inception to 4 March 2025. The search strategy was based on keywords using Boolean operators (adapted from the databases) based on a PICOS (population, intervention, comparison, outcome, and study design) strategy: ‘human’, ‘adult’, ‘healthy individuals’, ‘athletes’, ‘patients’, ‘high-intensity interval training’, ‘interval training’, ‘sprint interval training’, ‘HIIT’, ‘SIT’, ‘moderate-intensity continuous training’, ‘MICT’, ‘exerkines’, ‘myokines’, ‘cytokines’, ‘neuroproteins’, ‘controlled clinical trial’. As an example, the PubMed search included the following terms (‘high-intensity interval training’ OR ‘HIIT’ OR ‘sprint interval training’ OR ‘SIT’) AND (‘myokines’ OR ‘exerkines’ OR ‘cytokines’). The full search strategy for each database is available in the Supplementary Material (Supplementary Table S2).

The identified results were then transferred to the Mendeley Reference Manager tool, which was used in the later stages of the review.

### 2.2 Eligibility criteria

All articles were initially screened based on their titles and abstracts by ZJ and SK to ensure consensus. Articles that were deemed eligible were assessed in full text before being included. The agreement between the authors was assessed using Cohen’s kappa coefficient, which demonstrated a value of 0.82, suggesting nearly perfect agreement. No disagreements arose that required discussion with the third reviewer (RL). The selection criteria for the studies in this review were determined based on the Population, Intervention, Comparison, Outcomes, and Study (PICOS) framework:

Population: humans with a mean age of ≥18 years old; regardless of their health status,
Intervention: HIIT - both acute (single exercise session) and chronic effects (training) with at least two limbs trained.
  ➢Exercise intensity, as one of the criteria for HIIT, was adopted [(Coates et al., 2023; Boyne et al., 2013; Weston et al., 2014)]:
  For healthy adults:
    •SIT as “all-out” bursts, or
    •≥85% of maximal/peak heart rate (HR_max/peak_) or maximal velocity or velocity corresponding to VO_2max_, or
    •≥80% VO_2max/peak_ or peak power output (PPO), or W_max_ or
    •17 in (6–20) Borg’s scale.
  For diseased adults:
    •SIT as “all-out” bursts, or
    •≥85% HR_max/peak_ or maximal velocity or velocity corresponding to VO_2max_, or
    •≥60% of heart rate reserve (HRR) for cardiovascular patients
    •≥80% VO_2max/peak_ or PPO, or W_max_, or
    •or 7–8 in Borg’s (0–10) or 14–17 (6–20) scales for vigorous intensity.
  For aged adults:
    •≥75% HR_max_, or
    •≥60% of heart rate reserve (HRR)
    •≥80% VO_2max/peak_ or PPO, or W_max_, or
    •7–8 in Borg’s (0–10) or 14–17 (6–20) scales for vigorous intensity.
  To minimize confounding effects, this study excluded:
    •Hybrid training,
    •High-intensity circuit/resistance training,
    •Plyometrics, and other modalities,
    •Combined with supplementation and medication protocols,
    •Combined with other interventions.
Comparison: MICT or low intensity training (LIT) or CON or normal or usual care;
  •HIIT or SIT interventions without at least 1 comparator were excluded.
Outcome: at least one biomarker has been analyzed (myokine, exerkine, cytokine, neuroprotein, tryptophan metabolite) in plasma or serum samples.
  Studies were excluded from analysis if:
    •Non-blood specific tissue content (brain/skeletal muscle *etc.*) concentration have been assessed
    •Receptor levels have been determined.
Studies: Clinical trials, cohort studies, and longitudinal studies that satisfied the predetermined inclusion criteria.

### 2.3 Data extraction

The included papers were reviewed in full text and the main data were extracted and combined in an *ad hoc* table, which was designed as follows: 1) study characteristics (author’s name, date of publication), 2) participants (sample size, proportion of men and women, age, health status), 3) intervention (exercise protocol used with information about its duration (if applicable), volume and intensity), 4) comparison (information about comparative conditions (exercise protocol if applicable, or control) with information about volume and intensity), 5) outcome (regarding comparator or information about both protocols, timing of blood sample collection (post), and sample type).

These data were independently extracted by two reviewers (ZJ and SK). A third reviewer (RL) was appointed to mediate between disagreements between reviewers. Studies that did not meet the eligibility criteria are listed in the Supplementary Material (Supplementary Table S3).

### 2.4 Risk of bias assessment

The reviewers (ZJ and SK) independently assessed the risk of bias of the studies using the Cochrane Risk of Bias Tool for Randomized Clinical Trials (RoB 2.0) (Higgins et al., 2019). If there were any discrepancies, a third reviewer (RL) was asked for his opinion in order to resolve the disagreement. The risk of bias tool covers five domains and contains the following biases: randomization process, deviations from intended interventions, missing outcome data, measurement of the outcome, and selection of the reported result. Each of these domains can be categorised as ‘low risk of bias’, unclear risk of bias, i.e., ‘some concerns’, and ‘high risk of bias’. Low risk of bias was determined if all domains had an unclear risk of bias. If a minimum of one domain had an unclear risk of bias or high risk of bias, then the overall judgement was ‘some concerns’ or ‘high risk of bias’, respectively.

## 3 Results

### 3.1 Study selection and characteristics

**FIGURE 2:**
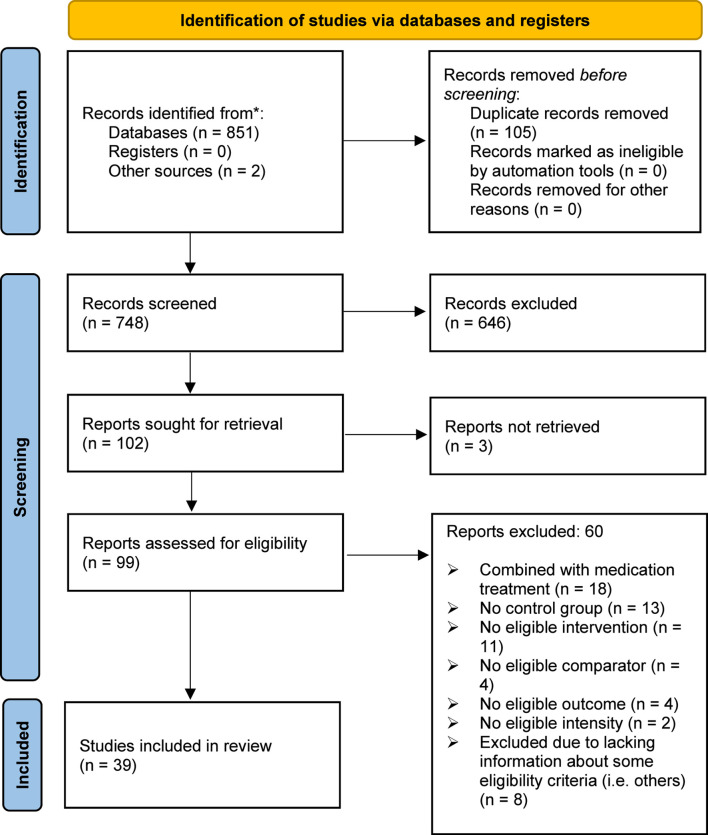
Study selection flow diagram.

Of the 853 studies identified in databases and other sources, 105 were removed due to duplicate data. Following the screening of 748 records, 102 were sought for retrieval, of which 99 underwent full-text evaluation. Ultimately, 39 studies met the eligibility criteria. The selection process is presented in Figure 2.

Of the 39 studies analysed, 14 focused on the acute effects, 22 on the chronic effects, and three on both acute and chronic effects. These studies are summarised in Tables 1–3, respectively.

**TABLE 1:** Acute effects of HIIT on exerkines.

Study characteristics	Participants			Intervention		Comparison	Outcome (regarding comparator)	
	n (male/female)	Age (y) ± SD	Health status	Protocol (duration)	Volume and intensity (reps × time/rest)	Protocol (Volume and Intensity)	Timing (post) and sample type (plasma/serum)	Outcome
Acute								
Boyne et al. (2019)	16 (9/7)	57.4 ± 9.7	Ambulatory adults with chronic stroke	a) HIIT on a treadmill b) HIIT on a stepper	30″/30–60″ in total 20′ >60% HRR/passive rest	MCE on a treadmill 20′, 45% HRR	i.a serum	a) ↑BDNF b) ↔BDNF
Boyne et al. (2020)	16 (9/7)	57.4 ± 9.7	Ambulatory adults with chronic stroke	a) HIIT on a treadmill b) HIIT on a stepper	30″/30–60″ in total 20′ >60% HRR/passive rest	MCE on a treadmill 20′, 45% HRR	i.a serum	a) ↑IGF-1, VEGF b) ↑IGF-1 ↔ Cortisol in both groups
Hoekstra et al. (2017)	12 (12/0)	22.5 ± 3.3	Recreationally active adults	HIIT on an arm-crank cycle ergometer	1′/1′ for 20′ 200% PO/active rest	a) CON 30′ 80% PO b) CAD 30′ 80% PO with alternating cadence	i.a., and 2 h post plasma	Increased IL-6 in all groups i.a. and 2 h post-exercise Ns between groups
Kaspar et al. (2016)	7 (1/6)	20.9 ± 0.9	Healthy untrained adults	SIT on a cycle ergometer	6 × 30″/4′ “all-out”/active or passive rest	ET on a cycle ergometer 45′ 62.5% HR_max_	30′ and 2 days post; plasma	↔ IGF-1, IL-1*β*, IL-6, IL-10
Kujach et al. (2019)	36 (36/0)	∼21	Healthy adults	SIT on a cycle ergometer	6 × 30″/4′30″ “all-out”/passive rest	Inactive control	i.a., and 1 h post serum	i.a.:↑BDNF, VEGF ↔ IGF-1 1 h post: ↑BDNF, VEGF
Marquez et al. (2015)	26 (26/0)	28 ± 1	Healthy adults	HIIT on a cycle ergometer	10 × 1′/1′ 90% W_max_/active rest	CON on a cycle ergometer 20′ 70% W_max_	i.a serum	↑BDNF
Peake et al. (2014)	10 (10/0)	33.2 ± 6.7	Well-trained cyclists and triathletes	HIIT on a cycle ergometer	10 × 4′/2′ ∼80% VO_2max_/active rest	MOD on a cycle ergometer 60′ ∼65% VO_2max_	i.a plasma	↑Cortisol Increased IL-6 after both HIIT and MOD exercises
Proschinger et al. (2023)	24 (12/12)	29.7 ± 4.3	Recreationally active runners	HIIT on a treadmill	6 × 3′/3′ 90% VO_2peak_/active rest	MCE on a treadmill 50′ 70% VO_2peak_	i.a., and 1 h post serum	i.a.: Increased IL-6 after both exercises; 1 h post: Increased IL-10 after both exercises
Rioux et al. (2024)	52 (18/34)	a) 30.0 ± 8.7 b) 37.8 ± 8.1	a) Adults with normal weight/no obesity (n = 26; 18 F) b) Adults with overweight/obesity (n = 26; 16 F)	HIIT on a cycle ergometer	2′after every 5′ (total 35′) 85%–90% HRR/active rest	a) MCE on a cycle ergometer 35′ 50%–55% HRR b) Inactive control	i.a plasma	↔Irisin
Rodriguez et al. (2018)	12 (12/0)	a) 22.58 ± 0.69 b) 25.54 ± 1.67	a) Adults with normal weight b) Adults with obesity	HIIT on a treadmill	4 × 4′/3′ 80%–90% VO_2max_/active rest	CME on a treadmill 38′ 50%–60% VO_2max_	i.a serum	a) ↔BDNF b) ↑BDNF
Rohnejad and Monazzami (2023)	12 (12/0)	∼45.0–47.8	Adults with overweight	HIIT (running)	4x (4 × 30″/30″)/5′ 100%MAV/50%MAV/passive rest	Inactive control	1 h post; serum	↔Cortisol ↑IL-6
Slusher et al. (2018)	13 (13/0)	23.62 ± 1.06	Healthy adults	SIT on a cycle ergometer	10 × 20″/10″ “all-out”/active rest	Inactive control	i.a plasma and serum	↑BDNF in serum, but not in plasma
Tsai et al. (2021)	21 (10/11)	60.62 ± 4.96	Healthy adults	HIIT on a cycle ergometer	1′/2′ (total 24′) 70%–75% HRR/active rest	a) MCE on a cycle ergometer 24′, 50%–55% HRR b) Inactive control	i.a serum	a) Increased BDNF in HIIT and MICE, ns between groups b) ↑BDNF, Irisin
Wahl et al. (2014)	12 (12/0)	24.7 ± 3.4	Healthy triathletes/cyclists	a) 4 × 4′ b) 4 × 30″ HIIT on a cycle ergometer	a) 4 × 4′/3′ 90–95%PPO/active rest b) 4 × 30″/7′30″ “all-out”/active rest	HVT on a cycle ergometer 120′ 55% PPO	i.a serum	a) ↑VEGF b) ↑VEGF

Abbreviations: BDNF, brain-derived neurotrophic factor; CAD, moderate-intensity exercise with changes in cadence; CME, continuous moderate exercise; CON, continuous exercise group; HIIT, high-intensity interval training; HRR, heart rate reserve; HVT, high volume training; IGF-1, insulin-like growth factor-1; IL-1β, interleukin-1 beta; IL-6, interleukin-6; IL-10, interleukin-10; i.a., immediately after; MAV, maximum aerobic velocity; MCE, moderate continuous exercise; MOD, moderate intensity training; PO, power output; PPO, peak power output; SIT, sprint interval training; VEGF, vascular endothelial growth factor.

**TABLE 2:** Chronic effects of HIIT on exerkines.

Study characteristics	Participants			Intervention		Comparison	Outcome (regarding comparator)	
	n (male/female)	Age (y) ± SD	Health status	Protocol (duration)	Volume and intensity (reps × time/rest)	Protocol (Volume and Intensity)	Timing (post) and sample type (plasma/serum)	Outcome
Chronic								
Alizadeh and Safarzade (2019)	20 (20/0)	18.0 ± 1.5	Adults with overweight	SIT: shuttle running (6 weeks; 3/w)	4–6 × 30″/30″ “all-out”/passive rest	Inactive control	24–28h; serum	↑IL-4 ↑IL-13
Allen et al. (2017)	55 (20/35)	49.2 ± 6.1	Adults with overweight	a) HIIT on cycle ergometer b) PIST on cycle ergometer (9 weeks; 3/w)	a) 4–8 × 20–30″/3′10″-5′; “all-out”/passive rest b) 10–20 × 10″/1′30″-3′10″; “all-out”/active rest	Inactive control	nd plasma	a) ↔TNFα b) ↔TNFα
Banitalebi et al. (2019)	52* (0/52) (*28 included in the analysis)	55 ± 6	Adults with overweight and T2D	SIT on cycle ergometer (10 weeks; 3/w)	4 × 30″/2′ “all-out”/active rest	Inactive control	48h; serum	↔ FGF-21, IL-6, IL-15, Irisin, SPARC
Barry et al. (2018)	33 (5/28)	∼43.8–50.4	Adults with obesity	HIIT: treadmill or outdoor walking, or cycle ergometer (2 weeks; 5/w)	4–10 × 1′/1′ ∼90% HR_peak_/active rest	MICT: treadmill or outdoor walking or cycle ergometer 20–50′, ∼65% HR_peak_	∼72h; plasma	↔IL-6, IL-10, TNFα
Coletta et al. (2021)	33 (0/33)	∼62.6–66.1	Postmenopausal females with overweight or obesity	HIIT on a treadmill; (12 weeks; 3/w)	4 × 4′/3′; 90%–100% HR_peak_/active rest	a) MICT on a treadmill 41′ 60%–70% HR_peak_ b) Usual care	3–5 days; plasma	↔IL-6, IL-7, IL-15, Irisin
Cooper et al. (2016)	62 (62/0)	49.5 ± 5.8	Adults with overweight	a) P-SIT on cycle ergometer b) A-SIT on cycle ergometer (12 weeks; 3/w)	a) 4–10 × 30″/3′ passive rest b) 4–10 × 30″/3′ active rest	a) ET on a cycle ergometer 50–60′, ∼80%–85% HR_max_ b) Inactive control	nd plasma	↔IL-6, IL-10, TNFα
de Lima et al. (2022)	25 (25/0)	∼40	Adults with overweight	HIIT: shuttle running (8 weeks; 3/w)	7–1 × (10 × 20 m)/1′ 85%–100% V_max_/passive rest	MICT: track running 3500–5000 m 60%–75% V_max_	24h; serum	↔BDNF Increase in both groups
Farrow et al. (2024)	27 (13/14)	46 ± 9	Chronic spinal cord injury between T2 and L5; > 1-year post-injury	HIIT on an arm-crank cycle ergometer (6 weeks; 4/w)	10 × 1′/1′ 80–90 HR_peak_/active rest	Inactive control	nd nd	↔Adiponectin, Leptin
Gao et al. (2023)	33* (33/0) (*23 included in the analysis)	46.2 ± 4.6	Adults with obesity and metabolic syndrome	HIIT on a treadmill (12 weeks; 3/w)	4 × 4′/3′ 90% HR_peak_/active rest	Inactive control	nd plasma	↑Adiponectin
Gerosa-Neto et al. (2016)	32 (both sexes; nd regarding quantity)	46.4 ± 10.1	Adults with overweight and obesity	a) 1-bout HIIT on a treadmill b) HIIT on a treadmill (16 weeks; 3/w)	a) 1 × 4′ 90% HR_max_ b) 4 × 4′/3′ 90% HR_max_/active rest	CONT on a treadmill 30′ 70% HR_max,_ (5/w)	nd plasma	a) ↔IL-6, IL-10, TNFα b) ↓IL-6; ↑TNFα; ↔IL-10, Decreased Adiponectin in all groups
Haghighi et al. (2022)	30* (30/0) (*20 included in the analysis)	36.56 ± 3.33	Adults with overweight and obesity	HIIT: shuttle running (8 weeks; 3/w)	5–8 × 30″/30″ 85%–95% HR_max_/nd	Inactive control	48h; serum	↑Irisin, FGF-21
Heiston et al. (2020)	28 (6/22)	61 ± 8.4	Adults with overweight and obesity	HIIT on a cycle ergometer (12 exercise bouts within 2 weeks)	3′/3′ for 60′ 90% HR_peak_/active rest	CONT on a cycle ergometer 60′ 70% HR_peak_	nd plasma	↑Adiponectin Decreased Leptin in both groups
Hovanloo et al. (2013)	16 (8/8)	∼22–25	Recreationally active adults	SIT on a cycle ergometer (2 weeks; 3/w)	4–6 × 30″/4′ “all-out”/passive rest	CET on a cycle ergometer 90–120′, 65% VO_2max_	48h; serum	↔IL-6, IL-10
Hovsepian et al. (2021)	30* (0/30) (*26 included in the analysis)	20.53 ± 1.50	Adults with overweight and obesity	All extremity HIIT on a cycle ergometer (10 weeks; 4/w)	4 × 4′/3′ 85%–90% HR_max_/active rest	Inactive control	48h; serum	↑Adiponectin ↔FGF-21
Javelle et al. (2021)	53 (17/36)	∼29–31	Emotionally impulsive adults	HIIT on a cycle ergometer (8 weeks; 3/w)	4 × 4′/3′ 85%–95% HR_max_/active rest	Active control - stretching	>36h; serum	↓IL-6 ↓QA/KYN ↑KYNA/QA
Kang et al. (2024)	52 (52/0)	63.4 ± 7.1	Patients with prostate cancer on active surveillance	HIIT on a treadmill (12 weeks; 3/w)	5–8 × 2′/2′ 85%–95% VO_2peak_/active rest	Usual care	≥72; plasma	↓IGF-1 ↔Adiponectin, Leptin
Kordi et al. (2013)	22 (0/22)	∼18	Sedentary adults	HIIT: shuttle running (6 weeks; 3/w)	4–6x30″/30″ “all-out”/nd	Inactive control	48h; plasma	↑Adiponectin
Lucibello et al. (2020)	46 (17/29)	19.9 ± 2.2	Adults with a low physical activity level	HIIT on cycle ergometer (9 weeks; 3/w)	10 × 1′/1′ ∼90–95 HR_max_/active rest	Inactive control	nd serum	↔IL-1β, IL-6, TNF-α
Middelbeek et al. (2021)	22 (22/0)	48 ± 5	Sedentary adults	SIT on a cycle ergometer (2 weeks; 3/w)	6 × 30″/4′ “all-out”/nd	MICT on a cycle ergometer 40–60′, 60% VO_2peak_	48h; serum	↔IL-8, TNFα Increased Klotho in MIT; Reduced IL-6 and Leptin in both groups
Rentería et al. (2020)	17 (0/17)	22 ± 1	Healthy adults	HIIT on a cycle ergometer (4 weeks; 3/w)	3–5 × 30″/4′ 80% MAP/active rest	Inactive control	48h; serum	↑BDNF
Yang et al. (2024)	20 (0/20)	∼21–22	Adults with overweight	HIIT on a treadmill (10 weeks; 3/w)	5 × 4′/3′ 85% VO_2max_/active rest	COP on a treadmill 45′ 60.15% VO_2max_ Treadmill running	within 1 week plasma	Increased IL-6 in HIIT and COP, ns between groups
Zhang et al. (2024)	42* (0/42) (*28 included in the analysis)	22.33 ± 2.18	Sedentary adults	HIIT on a cycle ergometer (8 weeks; 3/w)	2–3 × (12–15 × 30″/10″)/5′ “all-out”/nd/walking allowed	Inactive control	72h; plasma	↔IL-8, Leptin; Reduced TNFα in both groups

Abbreviations: A-SIT, sprint interval training with active rest; BDNF, brain-derived neurotrophic factor; CET, continuous endurance training; CONT, moderate-continuous exercise training; COP, crossover point exercise training; ET, endurance training; FGF-21, fibroblast growth factor-21, HIIT, high-intensity interval training; IGF-1, insulin-like growth factor-1; IL-1β, interleukin-1 beta; IL-4, interleukin-4; IL-6, interleukin-6; IL-7, interleukin-7; IL-8, interleukin-8; IL-10, interleukin-10; IL-13, interleukin-13; IL-15, interleukin-15; KYN, kynurenine; KYNA, kynurenic acid; MICT, moderate-intensity continuous training; MIT, moderate intensity training; P-SIT, sprint interval training with passive rest; PIST, prolonged intermittent sprint training; QA, quinolinic acid; SIT, sprint interval training; SPARC, secreted protein acidic and rich in cysteine; T2D, type 2 diabetes; TNFα, tumour necrosis factor alpha.

**TABLE 3:** Acute and chronic effects of HIIT on exerkines.

Study characteristics	Participants			Intervention		Comparison	Outcome (regarding comparator)	
	n (male/female)	Age (y) ± SD	Health status	Protocol (duration)	Volume and intensity (reps x time/rest)	Protocol (Volume and Intensity)	Timing (post) and sample type (plasma/serum)	Outcome
Acute and chronic								
Elmer et al. (2016)	12 (12/0)	21.4 ± 1.6	Sedentary-to-physically-inactive adults	a) Acute HIIT on a treadmill b) HIIT on a treadmill (8 weeks; 3/w)	12 × 1′/1′ 90%–110% vVO_2max_/active rest	ET 24′ 70%–80% vVO_2max_	a) within 2 min b) 72–96 h plasma	a) ↔IL-6 b) ↔IL-6
Richards et al. (2010)	31 (9/22)	23–29	Healthy adults	a) Acute SIT on a cycle ergometer b) SIT on a cycle ergometer (2weeks; 3/w)	a) 4 × 30″/4′ b) 4–7x30″/4′ “all-out”/nd	Inactive control	72h; plasma	a) ↔Adiponectin b) ↔Adiponectin
Sasimontonkul and Sirivarasai (2024)	22 (0/22)	∼40	Adults with overweight	a) Acute HIIT on a treadmill b) HIIT on a treadmill (16 weeks; 3/w)	1′/2′ (40′ in total) 80%–90% HRR/active rest	Inactive control	a) i.a b) i.a. last session serum	a) ↑IL6, ↔Adiponectin, Leptin (last session) b) ↑Adiponectin, ↔IL-6, Leptin

Abbreviations: ET, endurance training; HIIT, high-intensity interval training; IL-6, interleukin-6; SIT, sprint interval training.

### 3.2 Acute effects of HIIT on exerkines

All the studies analysing the acute exercise-related effects on the release of selected exerkines were published between 2014 and 2024, and these are shown in Table 1.

#### 3.2.1 Participants

The studies included 269 participants, 71% of whom were male. Eight studies were conducted with male only (Hoekstra et al., 2017; Kujach et al., 2019; Marquez et al., 2015; Peake et al., 2014; Rodriguez et al., 2018; Rohnejad and Monazzami, 2023; Slusher et al., 2018; Wahl et al., 2014), and six with both male and female adults (Boyne et al., 2019; Boyne et al., 2020; Kaspar et al., 2016; Proschinger et al., 2023; Rioux et al., 2024; Tsai et al., 2021). The age ranged from 20.9 to 60.62 years. Eight studies were conducted in healthy or regularly exercising adults (Hoekstra et al., 2017; Kaspar et al., 2016; Kujach et al., 2019; Marquez et al., 2015; Peake et al., 2014; Proschinger et al., 2023; Slusher et al., 2018; Wahl et al., 2014), two with adults with overweight, and obesity (Rioux et al., 2024; Rodriguez et al., 2018), one in adults with overweight (Rohnejad and Monazzami, 2023), two in adults with chronic stroke (Boyne et al., 2019; Boyne et al., 2020), and one in healthy, older adults (Tsai et al., 2021).

#### 3.2.2 Intervention and comparison

Of the 14 included studies, 10 were based on classical HIIT (Boyne et al., 2019; Boyne et al., 2020; Hoekstra et al., 2017; Marquez et al., 2015; Peake et al., 2014; Proschinger et al., 2023; Rioux et al., 2024; Rodriguez et al., 2018; Rohnejad and Monazzami, 2023; Tsai et al., 2021), three implemented SIT “all-out” bursts (Kaspar et al., 2016; Kujach et al., 2019; Slusher et al., 2018), and one used both HIIT and SIT (Wahl et al., 2014). Ergometer cycling was conducted in eight studies (Kaspar et al., 2016; Kujach et al., 2019; Marquez et al., 2015; Peake et al., 2014; Rioux et al., 2024; Slusher et al., 2018; Tsai et al., 2021; Wahl et al., 2014), while one study used an arm crank-based ergometer cycling (Hoekstra et al., 2017). Three studies implemented protocols based on running or walking (Proschinger et al., 2023; Rodriguez et al., 2018; Rohnejad and Monazzami, 2023). Additionally, two studies (Boyne et al., 2019; Boyne et al., 2020) used specific HIIT modalities performed on a treadmill or stepper.

The duration of the exercise protocols varied. Six studies included work bouts lasting up to 30 s (Boyne et al., 2019; Boyne et al., 2020; Kaspar et al., 2016; Kujach et al., 2019; Rohnejad and Monazzami, 2023; Slusher et al., 2018), three included 1-min bouts (Hoekstra et al., 2017; Marquez et al., 2015; Tsai et al., 2021), one included 2-min bouts (Rioux et al., 2024), and one included 3-min bouts (Proschinger et al., 2023). Four-minute work bouts were applied in two studies (Peake et al., 2014; Rodriguez et al., 2018), while in one study both 30 s and 4-min bouts were applied (Wahl et al., 2014).

Rest durations between work bouts varied substantially across the protocols. For HIIT protocols, recovery bout intervals most commonly ranged from 1 to 3 min, typically performed as active rest (Hoekstra et al., 2017; Marquez et al., 2015; Peake et al., 2014; Proschinger et al., 2023; Rioux et al., 2024; Rodriguez et al., 2018; Tsai et al., 2021; Wahl et al., 2014). A 1:1 work-to-rest was applied in three studies (Hoekstra et al., 2017; Marquez et al., 2015; Proschinger et al., 2023). In exercise protocols in which longer work bout intervals were employed, such as 4-min bouts, generally incorporated 2–3 min of active recovery (Peake et al., 2014; Rodriguez et al., 2018; Wahl et al., 2014). For SIT protocols, the duration of the recovery bouts was generally longer to facilitate partial phosphocreatine replenishment and maintain performance capacity. Most studies adopted bouts ranging between four and 7.5 min of passive or active recovery following 30-s “all-out” work bouts (Kaspar et al., 2016; Kujach et al., 2019; Wahl et al., 2014). However, one study with an exercise protocol consisting of relatively short work bouts (e.g., 20-s sprints) also implemented brief 10-s recovery bouts (Slusher et al., 2018).

Of the 14 studies, only three included a non-exercise control group (Kujach et al., 2019; Rohnejad and Monazzami, 2023; Slusher et al., 2018), eight included a comparison group performing moderate-intensity continuous endurance training (Boyne et al., 2019; Boyne et al., 2020; Kaspar et al., 2016; Marquez et al., 2015; Peake et al., 2014; Proschinger et al., 2023; Rodriguez et al., 2018; Wahl et al., 2014), two studies included both exercise and non-exercise comparator groups (Rioux et al., 2024; Tsai et al., 2021), and one study included two exercise groups (Hoekstra et al., 2017).

#### 3.2.3 Outcome

In most studies, the analysed samples were collected immediately after the acute exercise session (12/14), with one study collecting samples 30 min after exercise (Kaspar et al., 2016), and 1 hour after exercise cessation (Rohnejad and Monazzami, 2023), respectively. In addition, nine studies analysed serum (Boyne et al., 2019; Boyne et al., 2020; Kujach et al., 2019; Marquez et al., 2015; Proschinger et al., 2023; Rodriguez et al., 2018; Rohnejad and Monazzami, 2023; Tsai et al., 2021; Wahl et al., 2014), four studies analysed plasma (Hoekstra et al., 2017; Kaspar et al., 2016; Peake et al., 2014; Rioux et al., 2024), and one study analysed both serum and plasma concentration of specific exerkines (Slusher et al., 2018).

BDNF concentrations in response to the study intervention were verified in six out of fourteen studies. In particular, higher serum BDNF concentrations were observed in three studies compared to a non-exercise control group, with two ones investigating SIT among healthy participants (Kujach et al., 2019; Slusher et al., 2018), and one investigating HIIT among older adults (Tsai et al., 2021). Furthermore, serum BDNF concentrations increased in four studies compared to an exercise control group: one study involved healthy participants (Marquez et al., 2015); one study found that the increase depended on the HIIT protocol used with post-stroke patients (Boyne et al., 2019); one study observed that the increase was only detectable among obese participants compared to normal-weight ones (Rodriguez et al., 2018); and one study noticed that the increase occurred in both the HIIT group and the exercise control group (Tsai et al., 2021).

VEGF concentrations were measured in three studies, all observed an increase either in response to the SIT protocol among healthy subjects (Kujach et al., 2019; Wahl et al., 2014), or in response to treadmill HIIT in post-stroke patients (Boyne et al., 2020). IGF-1 concentrations were also assessed in three studies, reporting that IGF-1 concentration remained unchanged in response to SIT among healthy subjects (Kaspar et al., 2016; Kujach et al., 2019), and increased in post-stroke subjects following a HIIT on a treadmill and stepper (Boyne et al., 2020).

Cortisol concentration was determined in three studies involving healthy, trained adults and adults with overweight. In two of these studies, no statistically significant change was observed in response to HIIT in post-stroke patients and adults with overweight (Boyne et al., 2020; Rohnejad and Monazzami, 2023), while in the third one (Peake et al., 2014) an increase in concentration was noticed after HIIT in well trained cyclist and triathletes. However, it is difficult to attribute these results to health status, or specific exercise characteristics, especially since the measurements were not taken at the same time points.

IL-6 levels were determined in five studies. Three of these studies showed increases independent of group allocation in healthy participants (HIIT vs. exercised control) (Hoekstra et al., 2017; Peake et al., 2014; Proschinger et al., 2023). One study showed an increase in adults with overweight in response to HIIT (Rohnejad and Monazzami, 2023). One study showed no change despite the use of SIT among healthy adults (Kaspar et al., 2016).

### 3.3 Chronic effects of HIIT on exerkines

A series of studies published between 2013 and 2024 analysed the chronic exercise-related effects on exerkine secretion. A detailed overview on the results is provided in Table 2.

#### 3.3.1 Participants

The studies included 688 subjects, 47% of whom were male (assuming that the number of subjects of both sexes was equal in one study (Gerosa-Neto et al., 2016). Seven studies included only one sex [male: (Alizadeh and Safarzade, 2019; Cooper et al., 2016; de Lima et al., 2022; Gao et al., 2023; Haghighi et al., 2022; Kang et al., 2024; Middelbeek et al., 2021); female: (Banitalebi et al., 2019; Coletta et al., 2021; Hovsepian et al., 2021; Kordi et al., 2013; Rentería et al., 2020; Yang et al., 2024; Zhang et al., 2024)]. Eight studies involved mixed samples with both sexes (Allen et al., 2017; Barry et al., 2018; Farrow et al., 2024; Gerosa-Neto et al., 2016; Heiston et al., 2020; Hovanloo et al., 2013; Javelle et al., 2021; Lucibello et al., 2020). Participants’ ages ranged from 18.0 to 66.1 years. Interestingly, 11 out of 22 studies were conducted among adults with overweight or obesity (Alizadeh and Safarzade, 2019; Allen et al., 2017; Banitalebi et al., 2019; Barry et al., 2018; Cooper et al., 2016; de Lima et al., 2022; Gao et al., 2023; Gerosa-Neto et al., 2016; Haghighi et al., 2022; Hovsepian et al., 2021; Yang et al., 2024), one with adults with psychological disorders (Javelle et al., 2021), and one with adults with a chronic spinal cord injury (Farrow et al., 2024). Six studies were conducted with healthy, active, or sedentary participants (Hovanloo et al., 2013; Kordi et al., 2013; Lucibello et al., 2020; Middelbeek et al., 2021; Rentería et al., 2020; Zhang et al., 2024). Three studies were conducted with older adults, two with overweight and/or obese participants (Coletta et al., 2021; Heiston et al., 2020), and one with patients with prostate cancer on active surveillance (Kang et al., 2024).

#### 3.3.2 Intervention and comparison

In 22 studies, the training duration of HIIT interventions ranged between 2 weeks (Barry et al., 2018; Heiston et al., 2020), ≥4 to ≤8 weeks (de Lima et al., 2022; Farrow et al., 2024; Haghighi et al., 2022; Javelle et al., 2021; Kordi et al., 2013; Rentería et al., 2020; Zhang et al., 2024), >8 to ≤12 weeks (Allen et al., 2017; Coletta et al., 2021; Gao et al., 2023; Hovsepian et al., 2021; Kang et al., 2024; Lucibello et al., 2020; Yang et al., 2024), and 16 weeks (Gerosa-Neto et al., 2016). In contrast, SIT-based studies were far less frequent, lasting 2 weeks (Hovanloo et al., 2013; Middelbeek et al., 2021), 6 weeks (Alizadeh and Safarzade, 2019), 10 weeks (Banitalebi et al., 2019), and 12 weeks (Cooper et al., 2016). Additionally, ten studies used ergometer cycling (Allen et al., 2017; Banitalebi et al., 2019; Cooper et al., 2016; Heiston et al., 2020; Hovanloo et al., 2013; Javelle et al., 2021; Lucibello et al., 2020; Middelbeek et al., 2021; Rentería et al., 2020; Zhang et al., 2024), one study used an arm-crank ergometer cycling (Farrow et al., 2024), and one study used all-extremity ergometer cycling (Hovsepian et al., 2021). Nine studies used treadmill walking/running or shuttle running (Alizadeh and Safarzade, 2019; Coletta et al., 2021; de Lima et al., 2022; Gao et al., 2023; Gerosa-Neto et al., 2016; Haghighi et al., 2022; Kang et al., 2024; Kordi et al., 2013; Yang et al., 2024), and another study allowed their participants to freely choose the type of exercise (Barry et al., 2018).

Exercise protocols with work bout durations of up to 30 s were used in ten studies (Alizadeh and Safarzade, 2019; Allen et al., 2017; Banitalebi et al., 2019; Cooper et al., 2016; Haghighi et al., 2022; Hovanloo et al., 2013; Kordi et al., 2013; Middelbeek et al., 2021; Rentería et al., 2020; Zhang et al., 2024). One-minute work bouts were used in three studies (Barry et al., 2018; Farrow et al., 2024; Lucibello et al., 2020), and two-to 4-min work bouts in seven studies (Coletta et al., 2021; Gao et al., 2023; Heiston et al., 2020; Hovsepian et al., 2021; Javelle et al., 2021; Kang et al., 2024; Yang et al., 2024), respectively. One study used both 1-min and 4-min work bouts (Gerosa-Neto et al., 2016), while one protocol was based on distance run, so its duration is unclear (de Lima et al., 2022).

In chronic HIIT and SIT interventions, the duration of recovery bouts showed some sizable variability, but the included studies tended to prioritize longer recovery durations to optimize adaptation and training compliance. For HIIT-based interventions, recovery bout intervals typically ranged from 1 to 3 min, with most protocols favouring active recovery (Barry et al., 2018; Farrow et al., 2024; Heiston et al., 2020; Kang et al., 2024; Lucibello et al., 2020). Programs employing 4-min work bouts consistently incorporated 3 min of active recovery (Coletta et al., 2021; Gao et al., 2023; Gerosa-Neto et al., 2016; Hovsepian et al., 2021; Javelle et al., 2021; Yang et al., 2024). For SIT-based programs, the recovery bout durations were substantially longer as compared to HIIT due to the supramaximal exercise intensity. Most studies used 3–4 min of passive or active recovery after 30-s “all-out” work bouts (Cooper et al., 2016; Hovanloo et al., 2013; Middelbeek et al., 2021).

Of the 22 studies, 11 included a non-exercise control group (Alizadeh and Safarzade, 2019; Allen et al., 2017; Banitalebi et al., 2019; Farrow et al., 2024; Gao et al., 2023; Haghighi et al., 2022; Hovsepian et al., 2021; Kordi et al., 2013; Lucibello et al., 2020; Rentería et al., 2020; Zhang et al., 2024), one included a usual care comparison group (Kang et al., 2024), one included an active control group [i.e., stretching (Javelle et al., 2021)], seven included a moderate-intensity continuous exercise control group (Barry et al., 2018; de Lima et al., 2022; Gerosa-Neto et al., 2016; Heiston et al., 2020; Hovanloo et al., 2013; Middelbeek et al., 2021; Yang et al., 2024), and two included mixed comparison groups [i.e., exercise with non-exercise (Cooper et al., 2016), exercise with usual care (Coletta et al., 2021)].

#### 3.3.3 Outcome

In ten studies, the blood used for further analysis was drawn up to 48 h after the last exercise session (Alizadeh and Safarzade, 2019; Banitalebi et al., 2019; de Lima et al., 2022; Haghighi et al., 2022; Hovanloo et al., 2013; Hovsepian et al., 2021; Javelle et al., 2021; Kordi et al., 2013; Middelbeek et al., 2021; Rentería et al., 2020), while in five studies, it was drawn ≥72 h after the last exercise session (Barry et al., 2018; Coletta et al., 2021; Kang et al., 2024; Yang et al., 2024; Zhang et al., 2024). In seven studies, the timepoint was not further specified (Allen et al., 2017; Cooper et al., 2016; Farrow et al., 2024; Gao et al., 2023; Gerosa-Neto et al., 2016; Heiston et al., 2020; Lucibello et al., 2020). Interestingly, plasma samples were analysed in half of the studies (11) (Allen et al., 2017; Barry et al., 2018; Coletta et al., 2021; Cooper et al., 2016; Gao et al., 2023; Gerosa-Neto et al., 2016; Heiston et al., 2020; Kang et al., 2024; Kordi et al., 2013; Yang et al., 2024; Zhang et al., 2024), serum samples in ten studies (Alizadeh and Safarzade, 2019; Banitalebi et al., 2019; de Lima et al., 2022; Haghighi et al., 2022; Hovanloo et al., 2013; Hovsepian et al., 2021; Javelle et al., 2021; Lucibello et al., 2020; Middelbeek et al., 2021; Rentería et al., 2020), and it remained unclear whether plasma or serum was analysed in one study (Farrow et al., 2024).

BDNF has been assessed in two studies. In one study involving healthy participants, an increase in serum BDNF levels was observed after HIIT (Rentería et al., 2020), whereas in another study involving participants with overweight, serum BDNF levels increased independently of group allocation (de Lima et al., 2022). Furthermore, three studies assessed serum (Banitalebi et al., 2019) and plasma (Coletta et al., 2021) irisin levels among participants with overweight and/or obesity, and two of these studies reported no change following SIT, or HIIT, respectively, while only one study reported an increase in its serum concentrations following HIIT (Haghighi et al., 2022).

In addition, only one study assessed plasma IGF-1 concentrations, and found them to be lower following HIIT compared to the usual care group in patients with prostate cancer on active surveillance (Kang et al., 2024).

Of the seven studies in which adiponectin was assessed, four documented an increase in its concentration, namely, three studies investigating adults with overweight and/or obesity (Gao et al., 2023; Heiston et al., 2020; Hovsepian et al., 2021), and one with sedentary adults (Kordi et al., 2013). Additionally, one study found that plasma adiponectin concentrations decreased independently of group allocation in adults with overweight and obesity (Gerosa-Neto et al., 2016), while another study involving adults with chronic spinal cord injury did not observe a significant change in adiponectin concentration (Farrow et al., 2024). Furthermore, plasma and serum leptin concentrations decreased irrespective of group allocation in adults with overweight and obesity (Heiston et al., 2020), and in sedentary ones (Middelbeek et al., 2021), respectively. Plasma leptin concentrations remained unchanged in sedentary adults (Zhang et al., 2024), patients with prostate cancer on active surveillance (Kang et al., 2024), and adults with chronic spinal cord injury (Farrow et al., 2024) in response to arm-crank cycle ergometer HIIT.

The profile of selected cytokines was assessed in thirteen studies (Alizadeh and Safarzade, 2019; Allen et al., 2017; Banitalebi et al., 2019; Barry et al., 2018; Coletta et al., 2021; Cooper et al., 2016; Gerosa-Neto et al., 2016; Hovanloo et al., 2013; Javelle et al., 2021; Lucibello et al., 2020; Middelbeek et al., 2021; Yang et al., 2024; Zhang et al., 2024); however, only IL-6, TNFα, and IL-10 are described here to illustrate the most significant changes (see Table 2 for further details). IL-6 concentration was assessed in ten studies. In six of these, its concentration was unchanged in adults with overweight and/or obesity (Banitalebi et al., 2019; Barry et al., 2018; Coletta et al., 2021; Cooper et al., 2016) and healthy ones (Hovanloo et al., 2013; Lucibello et al., 2020) after HIIT. In addition, one study observed a HIIT-related decrease in IL-6 concentration in emotionally impulsive adults (Javelle et al., 2021). In the remaining studies, the following IL-6 concentration changes were observed regardless of group allocation: an increase in adults with overweight (Yang et al., 2024), and a decrease in sedentary adults (Middelbeek et al., 2021). Notably, one study bouts in adults with overweight and obesity observed that the IL-6 responses dependent on the number of work bouts performed during the HIIT session, as no change were observed with one bout, while a reduction occurred after four (Gerosa-Neto et al., 2016).

TNFα was assessed in seven studies (Allen et al., 2017; Barry et al., 2018; Cooper et al., 2016; Gerosa-Neto et al., 2016; Lucibello et al., 2020; Middelbeek et al., 2021; Zhang et al., 2024). In five of these, TNFα concentrations remained unchanged, namely, in healthy adults in response to HIIT and SIT (Lucibello et al., 2020; Middelbeek et al., 2021), respectively, and in adults with overweight and/or obesity following HIIT (Allen et al., 2017; Barry et al., 2018; Cooper et al., 2016), and SIT (Allen et al., 2017; Barry et al., 2018; Cooper et al., 2016). In one study, a reduction in TNFα concentration was observed in sedentary adults, regardless of group allocation (Zhang et al., 2024). Conversely, one study found that TNFα concentration increased in response to HIIT with four bouts per session but remained unchanged throughout 16 weeks of one work bout per exercise session (Gerosa-Neto et al., 2016).

Interestingly, IL-10 concentrations were assessed in four studies, including either healthy adults (Hovanloo et al., 2013) or those with overweight and/or obesity (Barry et al., 2018; Cooper et al., 2016; Gerosa-Neto et al., 2016), with no evidence for statistically significant changes in response to HIIT or SIT.

### 3.4 Acute and chronic effects of HIIT on exerkines

All the studies analysing acute and chronic effects were published between 2010 and 2024 and are presented in Table 3.

#### 3.4.1 Participants

The acute and chronic effects of HIIT were examined in three studies involving a total of 65 participants (32% male). One study was conducted with male participants only (Elmer et al., 2016), one with female participants only (Sasimontonkul and Sirivarasai, 2024), and one with participants of both sexes (Richards et al., 2010). Participants’ ages ranged from 21.4 to approximately 40 years, and included healthy (Richards et al., 2010), sedentary-to-inactive (Elmer et al., 2016), adults and adults with overweight (Sasimontonkul and Sirivarasai, 2024).

#### 3.4.2 Intervention and comparison

Only one study implemented 30-s SIT bursts on a cycle ergometer over a period of 2 weeks (Richards et al., 2010). The remaining studies, involving 1-min HIIT bouts on a treadmill, lasted 8 weeks (Elmer et al., 2016), and 16 weeks (Sasimontonkul and Sirivarasai, 2024).

Two studies compared intervention protocols to a non-exercise control group (Richards et al., 2010; Sasimontonkul and Sirivarasai, 2024), while one study compared them to a moderate-intensity, continuous exercise control group (Elmer et al., 2016).

#### 3.4.3 Outcome

The time interval between exercise and blood sampling varied considerably. To investigate acute effects, blood sampling was performed immediately after exercise (Sasimontonkul and Sirivarasai, 2024), within 2 minutes (Elmer et al., 2016), or 72 h after the cessation of the exercise session (Richards et al., 2010). To examine the chronic effect, blood was drawn immediately after the last session (Sasimontonkul and Sirivarasai, 2024), 72 h after (Richards et al., 2010), or between 72 and 96 h after the last exercise session (Elmer et al., 2016). Outcomes were measured in both plasma (Elmer et al., 2016; Richards et al., 2010), and serum (Sasimontonkul and Sirivarasai, 2024).

Interestingly, only one study, conducted among adults with overweight, found that acute and chronic HIIT resulted in increased IL-6 and adiponectin concentrations, respectively (Sasimontonkul and Sirivarasai, 2024). Conversely, no changes in resting IL-6 or adiponectin levels were observed in sedentary-to-inactive and healthy adults (Elmer et al., 2016; Richards et al., 2010).

### 3.5 Risk of bias

In accordance with Risk of Bias tool (Higgins et al., 2019) two studies scored ‘low risk of bias’ (Proschinger et al., 2023; Coletta et al., 2021), two studies were considered ‘high risk of bias’ (Kordi et al., 2013; Rentería et al., 2020), and the remaining 35 were assessed as ‘some concerns’. Relative to the domain, most of the reviewed studies were rated as ‘high risk of bias’ in the selection of the reported results category, while the highest score awarded as ‘some concerns’ was in the category of randomisation process. The highest score as ‘low risk’ was rated in the domain measurement of the outcome (Supplementary Figure S1).

## 4 Discussion

### 4.1 HIIT in healthy populations

#### 4.1.1 BDNF and VEGF

To date, evidence of the efficacy of HIIT in improving the exerkine profile among healthy individuals is inconclusive. In this context, this systematic review adds to the literature that an increase in BDNF concentration has primarily been observed in studies with higher, often vigorous or “all-out” exercise intensity, particularly following acute SIT protocols (Kujach et al., 2019; Slusher et al., 2018), which is consistent with the observations of a recent meta-analysis (Rodríguez-Gutiérrez et al., 2024). In addition, VEGF concentrations also increased in response to either acute SIT or HIIT protocols (Kujach et al., 2019; Wahl et al., 2014). However, given that only one long-term study with a high risk of bias assessed BDNF concentrations (Rentería et al., 2020), and in light of the paucity of studies considering VEGF, particular attention should be paid in future research to advance our understanding of the potential transient increase in these exerkines following acute interventions, considering the role of potential moderators, such as exercise intensity, as high-intensity exercise typically triggers the release of BDNF from platelets, which is found in serum but not plasma (Slusher et al., 2018). Therefore, the long-term effects of HIIT (at least 12 weeks) on exerkines should be assessed, as our review provides evidence that this area is relatively under-researched. A significant source of variation in BDNF findings across the included studies is the biological matrix applied for analysis. Serum BDNF consistently increased following SIT or vigorous HIIT, whereas studies analysing plasma frequently reported no change, reflecting the influence of platelet degranulation on serum concentrations (Cho et al., 2012; Slusher et al., 2018). In addition, the timing of blood sampling varied substantially; studies collecting samples immediately post-exercise were more likely to detect increases in BDNF and VEGF than those sampling ≥30 min later, when values often approached baseline.

#### 4.1.2 Cytokines

In a homeostatic environment, a balance of pro- and anti-inflammatory cytokines should be present (Chauhan et al., 2021). An imbalance indicates the body’s defence response to inflammation, including that induced by acute exercise (Langston and Mathis, 2024). However, our findings are not fully consistent with current state of art, as the studies reviewed in this systematic review do not unequivocally demonstrate a significant change in inflammatory marker concentration in response to acute HIIT. Moreover, even in response to acute SIT, which should, according to theory, induce the greatest metabolic stress, no changes in the cytokine profile were observed (Kaspar et al., 2016). Nevertheless, the increase in IL-6 observed in some studies was independent of group allocation (HIIT/SIT vs. active controls). This suggests a response to increased metabolic stress, regardless of exercise intensity. Conversely, most of the studies analysed in this systematic review found no change in cytokine concentrations. These inconsistencies likely reflect a combination of protocol-related differences, including work-to-rest ratio, accumulated metabolic stress, and modality, as well as the timing of sampling, as IL-6 displays a rapid rise–fall profile with peak levels often occurring immediately after exercise (Proschinger et al., 2023). Studies collecting samples later (e.g., ≥30 min post-HIIT) were less likely to detect acute elevations.

Consequently, future studies should consider important factors that can influence the exerkine response to HIIT and SIT. These factors include but are not limited to the timing of blood sampling, the nutrition (i.e., glycogen storage) of the individual, and their initial body composition.

### 4.2 HIIT in diseased populations

Despite its well-documented safety, achieving a vigorous exercise intensity in the work bouts of HIIT can be challenging, especially for individuals with cardiovascular health issues (Carl et al., 2017; Taylor et al., 2020). In this context, data from HIIT studies in which individuals were taking specific medication is difficult to interpret because it requires additional efforts to disentangle the influence of the pharmaceutical treatment from that of HIIT. Therefore, this systematic review excludes those taking medication to examine only the effects of HIIT on the secretion of selected exerkines.

#### 4.2.1 BDNF

The secretion of BDNF, a key exerkine involved in neuroprotection (Knaepen et al., 2010), appears to be the main obstacle, given that BDNF release is established to be intensity-dependent (Fernández-Rodríguez et al., 2022). However, evidence, albeit limited, suggests that BDNF release is protocol-dependent. This is the case in post-stroke patients, as increased BDNF levels have been observed after an acute HIIT session on a treadmill but were seen after HIIT on a stepper (Boyne et al., 2019). This can be explained by the global and local involvement of skeletal muscles during treadmill and stepper sessions, respectively. In addition, an increase in BDNF levels following HIIT was also observed in obese but not in normal-weight individuals (Rodriguez et al., 2018). Differences in participant phenotype also help explain heterogeneous responses. For example, obese adults exhibited larger acute BDNF responses than normal-weight participants, likely due to higher baseline platelet counts and distinct inflammatory profiles (Rodriguez et al., 2018; Coban et al., 2005).

#### 4.2.2 VEGF and IGF-1

Similar observations have been made concerning VEGF and IGF-1, as an increase in their concentration is observed in adults following acute HIIT (Boyne et al., 2020). However, one chronic study indicates that IGF-1 decreased following chronic HIIT in cancer patients on active surveillance (Kang et al., 2024). This finding can be interpreted that the release of exerkines in response to HIIT depend on the health status. Moreover, among post-stroke adults, the larger BDNF and VEGF responses observed during treadmill-based HIIT compared with stepper-based HIIT may reflect greater recruitment of total muscle mass, which increases both metabolic stress and mechanical signalling (Boyne et al., 2019). IGF-1 is responsible for cell growth, proliferation, and maturation, and plays a key role in maintaining metabolic homeostasis (Al-Samerria and Radovick, 2023), but can be detrimental when not present in an appropriate concentration (Gubbi et al., 2018). Hypothetically, increases in circulating IGF-1 are undesirable during tumour development, which may explain the reduction in circulating IGF-1 following aerobic HIIT (Kang et al., 2024). Interestingly, IGF-1 is also involved in the regulation of the tissue insulin sensitivity and glycaemia, given that it has been reported to be associated with insulin resistance (Friedrich et al., 2012). However, the effects of HIIT on IGF-1 are not fully clear, since few studies, as shown by the present review, have investigated changes in IGF-1 concentrations in response to HIIT - even fewer among needy populations, such as overweight and obese individuals. Therefore, considering in future research the evaluation of the effects of HIIT on peripheral concentrations of proteins with extended metabolic effects, including IGF-1, adiponectin, and leptin, is important to gain a more comprehensive understanding of physiological responses triggered by HIIT. Such a more comprehensive understanding is a relevant perquisite to inform the application of HIIT as an intervention approach to improve health in specific populations.

#### 4.2.3 Adiponectin and leptin

Adiponectin and leptin can increase glucose uptake by skeletal muscle and also improve fatty acid oxidation (Friedrich et al., 2012). Adiponectin/leptin ratio dysregulation is observed in overweight, obesity, and metabolic syndrome (Stern et al., 2016). The functions of both proteins are opposing - adiponectin is anti-inflammatory, while leptin is pro-inflammatory (Frühbeck et al., 2018), and changes in their ratio are characteristic of greater fat accumulation (Funcke and Scherer, 2019). Interestingly, our findings are, at least partly, in line with those observations of a recent meta-analysis (Castela et al., 2023), which found that HIIT was associated with improvements in adiponectin. However, sources of heterogeneity that can explain such an observation might include the duration of the HIIT programme and the severity of obesity. Furthermore, based on the evidence in the literature, it appears plausible that engaging in HIIT for more than 12 weeks is more likely to reduce body fat, with more pronounced benefits on metabolic health (Khalafi and Symonds, 2020). However, our findings suggest that further research is required to explore the impact of moderators, such as age, body composition, and training duration (≥12 weeks), on the efficiency of HIIT to improve (cardiometabolic) health among individuals, who do not take specific medication, to address health consequences related to overweight and obesity.

### 4.3 HIIT in the aging population

Societal developments in the last century have culminated in a demographic change that is associated with higher life expectancy, which has become a growing challenge for the public health systems of industrialized societies (Batacan et al., 2017). More specifically, aging is accompanied by an increased prevalence of metabolic and neurological diseases, so it is, from a public health perspective, important to understand the molecular processes involved in their development (Cai et al., 2023; Lanctôt et al., 2024). Although there is evidence that physical activity, an umbrella term including planned and structured forms referred to as physical exercise and training (Caspersen et al., 1985), can counter some of the negative health effects of aging (Sun and Bao, 2024), the effectiveness of specific exercise modalities, such as HIIT, in slowing down aging and/or combating age-related health dysfunctions is not fully understood. Of the 39 studies reviewed, only four investigated the effect of HIIT on changes in metabolic and neuroactive exerkine concentrations among adults over 60 years of age (Tsai et al., 2021; Coletta et al., 2021; Heiston et al., 2020; Kang et al., 2024). Of these, only one included healthy older adults (Tsai et al., 2021). Interestingly, the lack of metabolic changes (Coletta et al., 2021; Kang et al., 2024), and the scarcity of information regarding specific exerkines (i.e., neuroproteins) (Tsai et al., 2021) does not allow to draw more firm conclusions, so that there remains a research gap of high practical relevance that needs to be addressed by future research.

For example, in real-world conditions, the inclusion criteria of single studies often exclude older adults who regularly take different types of medication from participating in HIIT interventions. Combined with the limited scope of the outcomes obtained from the four above articles, this indicates an urgent need to investigate the influence of HIIT on exerkines in the aging population.

Interpretation of the present findings must consider the substantial methodological and biological heterogeneity across studies, which likely contributed to the variability and occasional inconsistency in exerkine outcomes. First, differences in HIIT protocol including bout duration, work-to-rest ratio, recovery intensity, modality (cycling vs. treadmill vs. stepping), and the use of SIT *versus* HIIT may represent a primary source of variability (Laursen and Buchheit, 2019; Herold et al., 2025). The density of work and accumulated metabolic stress (e.g., lactate load) also differed meaningfully between protocols, influencing the underlying endocrine milieu and downstream exerkine secretion (Kujach et al., 2019).

Second, sample type (serum vs. plasma) introduced systematic differences, particularly for BDNF. Serum BDNF is strongly influenced by platelet degranulation, whereas plasma BDNF reflects circulating, non-platelet-derived fractions (Cho et al., 2012; Eckstrom et al., 2020). A study measuring serum reported a significant post-HIIT increase, whereas an analysis of plasma under similar exercise intensity found no clear effect (Slusher et al., 2018). Comparable discrepancies may apply to VEGF and selected cytokines, further complicating comparisons across studies.

Third, the timing of biomarker collection varied widely and played a decisive role in whether acute responses were detected. Most increases in BDNF, VEGF, and IL-6 occurred immediately post-exercise, whereas delayed sampling at 30–60 min or several hours post-exercise often resulted in attenuated or absent responses due to rapid return toward baseline (Kaspar et al., 2016; Proschinger et al., 2023). Chronic studies also differed substantially in post-training sampling windows (24 h to ≥96 h after the final session), affecting the ability to detect persistent adaptations *versus* transient responses (Coletta et al., 2021; Rentería et al., 2020).

Fourth, participant characteristics including age, adiposity, metabolic health, training status, and disease state likely moderated exerkine responses. Individuals with obesity demonstrated larger acute BDNF and adiponectin responses, potentially due to higher baseline platelet counts and altered inflammatory profiles (Rodriguez et al., 2018; Coban et al., 2005). Post-stroke adults exhibited differential responses depending on modality, with treadmill HIIT producing larger neurotrophic responses than stepper-based HIIT, possibly due to greater recruitment of total muscle mass (Boyne et al., 2019; Boyne et al., 2020). Older adults (>60 years) were underrepresented and showed more muted responses, consistent with age-related declines in neurotrophic and angiogenic signalling (Erickson et al., 2010; Grunewald et al., 2021). Differences in baseline inflammation between healthy, overweight, and diseased groups further influenced cytokine responsivity (Gao et al., 2023; Gerosa-Neto et al., 2016).

Finally, risk of bias patterns notably concerns regarding selective reporting, incomplete randomization, and unblinded outcome assessment add an additional layer of uncertainty. Although the overall methodological quality was acceptable, the predominance of “some concerns” ratings and the presence of several high-risk judgments limit the certainty of evidence, particularly for chronic adaptations where sample sizes were small and reporting insufficient. As a result, confidence in the stability and generalizability of many exerkine outcomes especially in clinical and older populations remains moderate to low. More rigorous methodological standards, including standardized load reporting, preregistered protocols, and complete reporting of outcomes, will be essential for strengthening the certainty of future HIIT-exerkine research.

### 4.3 Strengths and limitations

A strength of this systematic review was the applying of rigorous inclusion and exclusion criteria. For example, we minimized bias that would have arisen by including studies in which medication was not controlled, given that pharmacological treatment one of the main sources influencing exerkine responses (Yoshida et al., 2013; van Ginkel et al., 2016), was used as an exclusion criterion. This procedure reduced non-exercise-related heterogeneity in exerkine outcomes. With exercise intensity in the HIIT protocols tailored to the target population (i.e., healthy, older, or diseased adults), this systematic review also paves the way for further work to integrate participants’ internal load, operationalized through parameters such as heart rate or blood lactate concentrations. However, despite these strengths, focusing on individuals over 18 years of age does not allow generalization of findings to younger populations (e.g., children and adolescents). Also, we did not analyze potential sex differences in exerkine secretory responses. Some risk of bias remains in results concerning exerkine secretion (including IL-6, IGF-1, and cortisol), particularly arising from issues in the randomization process, which warrants cautious interpretation. Furthermore, we intentionally refrained from performing a meta-analysis because several exerkines of interest were not investigated in a sufficient number of studies to permit robust quantitative synthesis.

In contrast to previous reviews, which have typically focused on individual exerkines (e.g., only BDNF or only cytokines), specific populations (e.g., healthy young adults), or isolated protocol comparisons, this systematic review is the first to jointly consider and explicitly control for two major methodological confounders:

Insufficient or inconsistent exercise intensity definitions across populations, and
Pharmacological treatment, which strongly alters circulating exerkine profiles.

By applying strict population-specific intensity criteria and excluding all studies involving medications known to affect endocrine or inflammatory markers, this review synthesizes the HIIT–exerkine literature under conditions that more accurately isolate true exercise-induced signalling. No previous systematic review has combined these two methodological constraints while simultaneously evaluating acute and chronic responses across healthy, older, and diseased adults. This approach enhances internal validity and provides a more precise characterization of the exerkine response to HIIT than has previously been available.

### 4.4 Practical implications

The practical implementation of HIIT in real-world contexts requires of the consideration of aspect included but not limited to physiological potential to trigger a desired changes, and adherence (e.g., enjoyment of the training regime), with the latter is crucial especially to ensure longer-term adaptions (e.g., concerning cardiometabolic health). Evidence from our systematic review demonstrates that HIIT can elicit potent acute and chronic exerkine responses potentially benefiting a range of health dimensions (e.g., vascular, metabolic, and brain health), in both healthy and clinical populations. These findings provide, from a physiological perspective, a strong rationale for incorporating HIIT into health promotion and rehabilitation programs. With regard to practical implementation, however, the effectiveness of an intervention approach also depends on the participants adherence. Concerning HIIT, Ekkekakis and colleagues highlight that many extraordinary claims about HIIT’s advantage, especially as “time-efficient revolution”, are undermined by methodological weaknesses (e.g., underpowered trials, inflated Type I error risk, and selective interpretation of “comparable” results) (Ekkekakis et al., 2023a; Ekkekakis et al., 2023b; Ekkekakis and Biddle, 2023). Importantly, long-term adherence data from eight ≥12-month trials show that in real-world scenarios HIIT often do not confer substantially advantages over MICT, especially in unsupervised settings, as participants frequently reduce intensity below prescribed thresholds, and dropout rates are similar or higher than for MICT (Mueller et al., 2021). Furthermore, their interdisciplinary critique cautions that foundational pro-HIIT arguments, such as “lack of time” being the primary barrier or HIIT being universally safe and well tolerated - is often not supported by solid evidence.

Against this scepticism, Jung et al. provide countervailing evidence, challenging the assumption that HIIT triggers more negative affective responses, thus dooming adherence. Synthesizing multiple systematic reviews and meta-analyses, they report that HIIT is generally perceived as equally, and sometimes more, enjoyable than MICT, with supervised adherence exceeding 89% for both modalities. In unsupervised interventions, adherence falls for both (<69%), suggesting that the broader challenge lies not in HIIT *per se* but in sustaining any type of exercise without structured support. They further note that contextual factors such as music, social environment, and autonomy in protocol choice - can modulate affective responses to HIIT and self-efficacy, which are critical for long-term engagement. This aligns with the pragmatic recommendation that rather than framing HIIT vs. MICT as a zero-sum choice, implementation strategies should focus on tailoring interval formats to individual preferences, health status, and environmental opportunities (Jung et al., 2024).

Another critical but often overlooked factor in this debate is the role of “mode” when MICT is applied as the control condition. Mode encompasses the exercise type (e.g., cycling vs. running), structure (continuous vs. intermittent), and associated motor–cognitive demands, all of which can independently influence physiological, perceptual, and cognitive responses (Jiménez-Pavón and Lavie, 2017; Gentil and Del Vecchio, 2017). When HIIT and MICT differ not only in intensity but also in mode, observed between-group differences risk being confounded by these mode-specific effects rather than intensity *per se*. Even within the same modality (e.g., cycling), cadence variability, muscle recruitment patterns, and repeated accelerations in HIIT introduce unique neuromuscular and perceptual stimuli absent in MICT. These differences can alter cardiovascular strain, metabolic cost, and affective responses - potentially impacting both acute adaptations and long-term adherence. If not carefully controlled, such differences could lead to misleading conclusions about HIIT’s acceptability, adherence potential, or cognitive benefits (Herold et al., 2021).

Therefore, HIIT and MICT comparisons should, wherever possible, use identical exercise modalities and match mechanical and coordinative demands as well as energy expenditure. Where perfect matching is not feasible, we recommend that mode differences must be explicitly acknowledged and analyzed, with secondary measures (e.g., internal load indices, perceptual responses) used to assess their potential influence. This entails detailed reporting of both external load (e.g., power output, velocity) and internal load (e.g., heart rate, lactate, rate of perceived exertion, energy expenditure), as well as documenting movement patterns and any added motor–cognitive elements. Without such methodological precision, the field risks over- or underestimating the “true” role of exercise intensity, reinforcing misconceptions about HIIT’s unique benefits or limitations (Herold et al., 2021).

Finally, a pragmatic way to deal with the issue surrounding HIIT is to frame it not as a universal replacement for moderate-intensity intervention approaches, but as an option that, when appropriately individualized, and supported, can play a valuable role in a person-centred, and diversified exercise prescriptions.

## 5 Conclusion

In this systematic review, we provide the first synthesis of HIIT-induced exerkine responses that simultaneously accounts for two major methodological confounders exercise intensity adequacy and pharmacological treatment by applying population-specific intensity criteria and excluding all studies involving medications known to modify endocrine or inflammatory markers. This approach reduces non-exercise–related bias and enables a more accurate assessment of acute and chronic exerkine dynamics in healthy, older, and diseased adults.

Across the available evidence, acute serum BDNF and VEGF increase in an intensity-dependent manner, with the largest responses observed following SIT or vigorous HIIT protocols. However, chronic adaptations remain insufficiently studied, particularly for BDNF, VEGF, and other neurotrophic factors, limiting confidence in long-term mechanistic interpretations. Adiponectin responses appear more favorable in individuals with overweight or obesity, although findings for other metabolic or neuroprotective exerkines (e.g., IGF-1, irisin, cytokines) are inconsistent and often derived from small, heterogeneous samples. Given the predominance of studies rated as having “some concerns” or “high” risk of bias, the overall certainty of evidence, especially for chronic effects, is moderate to low, and conclusions should be interpreted with caution.

To strengthen future evidence, HIIT studies should justify the use of serum *versus* plasma matrices, incorporate isocaloric or energy-matched comparator protocols, and provide detailed information on the timing of blood sampling. Consistent reporting of internal load (e.g., blood lactate, heart rate, perceived exertion) and external load is essential to contextualize exerkine responses. Finally, there is a pressing need for longer-term (>12 weeks) interventions, more rigorous randomization and reporting practices, and greater inclusion of older adults and clinical populations to define the sustained impact of HIIT on neurotrophic, angiogenic, inflammatory, and metabolic exerkine pathways.

## Data Availability

The raw data supporting the conclusions of this article will be made available by the authors, without undue reservation.
