# Supplementary material for: Acute and chronic effects of high-intensity interval training on selected exerkine secretion in health, disease, and aging: a systematic review
Source: Front Physiol. 2026 Jan 23;16:1733269. doi: 10.3389/fphys.2025.1733269 (PMC12875996; doi:10.3389/fphys.2025.1733269)
Supplement: Supplementary file 1 [file DataSheet4.pdf]

Table S3. Excluded studies with reasons for exclusion.

Reference	Reason for exclusion
Aghaei Bahmanbeglou, N., Ebrahim, K., Maleki, M., Nikpajouh, A., & Ahmadizad, S. (2019). Short-Duration High-Intensity Interval Exercise Training Is More Effective Than Long Duration for Blood Pressure and Arterial Stiffness but Not for Inflammatory Markers and Lipid Profiles in Patients with Stage 1 Hypertension. *Journal of Cardiopulmonary Rehabilitation and Prevention*, *39*(1), 50–55. https://doi.org/10.1097/HCR.0000000000000377	Combined with medication treatment
Aghajani, M., Rahmati-Ahmadabad, S., Zamani, · Farhad, Behrouz Ghanbari, ·, & Azarbayjani, M.-A. (2021). The effects of high-intensity interval training and orlistat on selected adipokines and cytokines in obese women. *Springer*, *52*(1), 87–96. https://doi.org/10.1007/s12662-021-00749-z	No eligible intervention
Ahn, C., Ryan, B. J., Schleh, M. W., Varshney, P., Ludzki, A. C., Gillen, J. B., van Pelt, D. W., Pitchford, L. M., Howton, S. M., Rode, T., Hummel, S. L., Burant, C. F., Little, J. P., & Horowitz, J. F. (2022). Exercise training remodels subcutaneous adipose tissue in adults with obesity even without weight loss. *Journal of Physiology*, *600*(9), 2127–2146. https://doi.org/10.1113/JP282371	Combined with medication treatment
Aktaş, H. Ş., Uzun, Y. E., Kutlu, O., Pençe, H. H., Özçelik, F., Çil, E. Ö., Irak, L., Altun, Ö., Özcan, M., Özsoy, N., Aydın Yoldemir, Ş., Kalyon, S., Arman, Y., & Tükek, T. (2022). The effects of high intensity-interval training on vaspin, adiponectin and leptin levels in women with polycystic ovary syndrome. *Archives of Physiology and Biochemistry*, *128*(1), 37–42. https://doi.org/10.1080/13813455.2019.1662450	Excluded due to lacking information about some eligibility criteria (i.e. others)
Alizadeh, A. M., Isanejad, A., Sadighi, S., Mardani, M., kalaghchi, B., & Hassan, Z. M. (2019). High-intensity interval training can modulate the systemic inflammation and HSP70 in the breast cancer: a randomized control trial. *Journal of Cancer Research and Clinical Oncology*, *145*(10), 2583–2593. https://doi.org/10.1007/s00432-019-02996-y	Combined with medication treatment
Alizadeh, M., Shahrbanian, S., & Hackney, A. C. (2021). Comparison of the effects of 12 weeks of three types of resistance training (traditional, circular and interval) on the levels of neuregulin 4, adiponectin and leptin in non-athletic men with obesity. *Archivos de Medicina Del Deporte: Publicacion de La Federacion Espanola de Medicina Del Deporte*, *38*(6), 389. https://pmc.ncbi.nlm.nih.gov/articles/PMC9053093/	No eligible intervention
Andrews, S. C., Curtin, D., Hawi, Z., Wongtrakun, J., Stout, J. C., & Coxon, J. P. (2020). Intensity Matters: High-intensity Interval Exercise Enhances Motor Cortex Plasticity More Than Moderate Exercise. *Cerebral Cortex*, *30*(1), 101–112. https://doi.org/10.1093/cercor/bhz075	No eligible outcome

Avazpor, S., Fazel Kalkhoran, J., & Amini, A. (2016). Effect of 8 weeks of high intensity interval training on plasma levels of adiponectin and leptin in overweight nurses. *Novelty in Biomedicine*, *3*, 87–92. https://www.sid.ir/en/VEWSSID/J_pdf/5071320160304.pdf	Excluded due to lacking information about some eligibility criteria (i.e. others)
Avazpour, S., Kalkhoran, J., … K. A.-A. J. of, & 2020, undefined. (2020). The effect of two types of high-intensity interval training on serum value of GH and IGF-1 in overweight nurses. *Researchgate.Net*, *11*(4), 103135. https://doi.org/10.5812/asjsm.103135	Excluded due to lacking information about some eligibility criteria (i.e. others)
Bang-Kittilsen, G., Egeland, J., Ueland, T., Andersen, E., Bigseth, T. T., Holmen, T. L., Mordal, J., Holst, R., & Engh, J. A. (2023). The relationship between the brain-derived neurotrophic factor and neurocognitive response to physical exercise in individuals with schizophrenia. *Psychoneuroendocrinology*, *157*. https://doi.org/10.1016/j.psyneuen.2023.106356	No eligible comparator
Bartlett, D. B., Hanson, E. D., Lee, J. T., Wagoner, C. W., Harrell, E. P., Sullivan, S. A., Bates, L. C., Alzer, M. S., Amatuli, D. J., Deal, A. M., Jensen, B. C., MacDonald, G., Deal, M. A., Muss, H. B., Nyrop, K. A., & Battaglini, C. L. (2021). The Effects of 16 Weeks of Exercise Training on Neutrophil Functions in Breast Cancer Survivors. *Frontiers in Immunology*, *12*. https://doi.org/10.3389/fimmu.2021.733101	No eligible intervention
Bigseth, T. T., Engh, J. A., Andersen, E., Bang-Kittilsen, G., Egeland, J., Falk, R. S., Holmen, T. L., Mordal, J., Nielsen, J., Ueland, T., Vang, T., & Fredriksen, M. (2023). Alterations in inflammatory markers after a 12-week exercise program in individuals with schizophrenia—a randomized controlled trial. *Frontiers in Psychiatry*, *14*. https://doi.org/10.3389/fpsyt.2023.1175171	No eligible comparator
Bonet, J. B., Javierre, C., Guimarães, J. T., Martins, S., Rizo-Roca, D., Beleza, J., Viscor, G., Pagès, T., Magalhães, J., & Torrella, J. R. (2022). Benefits on Hematological and Biochemical Parameters of a High-Intensity Interval Training Program for a Half-Marathon in Recreational Middle-Aged Women Runners. *International Journal of Environmental Research and Public Health*, *19*(1). https://doi.org/10.3390/ijerph19010498	No eligible intervention
Candrawati, S., Huriyati, E., Sofro, Z. M., Rujito, L., Faza, A. N., Rohmawati, O. N., & Aqiilah, A. R. (2021). High-intensity Interval Training Improves Inflammatory Mediators in Obese Women: Based on the Study of the UCP2 Ala55Val Gene. *Open Access Macedonian Journal of Medical Sciences*, *9*(A), 871–875. https://doi.org/10.3889/oamjms.2021.6971	No control group
de Poli, R. A. B., Lopes, V. H. F., Lira, F. S., Zagatto, A. M., Jimenez-Maldonado, A., & Antunes, B. M. (2021). Peripheral BDNF and psycho-behavioral aspects are positively modulated by high-intensity intermittent exercise and fitness in healthy women. *Scientific Reports*, *11*(1). https://doi.org/10.1038/s41598-021-83072-9	No control group

Dehghani, K., & Mogharnasi, M. (2015). Effects of Ten Weeks of Aerobic Interval Training and Four Weeks Detraining on Plasma Adiponectin Level in Male Student Non-Athletes. *Zahedan Journal of Research in Medical Sciences 2015 17:10*, *17*(10). https://doi.org/10.17795/ZJRMS-2085	No eligible intensity
Devin, J. L., Hill, M. M., Mourtzakis, M., Quadrilatero, J., Jenkins, D. G., & Skinner, T. L. (2019). Acute high intensity interval exercise reduces colon cancer cell growth. *The Journal of physiology*, *597*(8), 2177–2184. https://doi.org/10.1113/JP277648	No control group
Dünnwald, T., Melmer, A., Gatterer, H., Salzmann, K., Ebenbichler, C., Burtscher, M., Schobersberger, W., & Grander, W. (2019). Supervised Short-term High-intensity Training on Plasma Irisin Concentrations in Type 2 Diabetic Patients. *INTERNATIONAL JOURNAL OF SPORTS MEDICINE*, *40*(3), 158–164. https://doi.org/10.1055/a-0828-8047	Combined with medication treatment
Durrer, C., Francois, M., Neudorf, H., & Little, J. P. (2017). Acute high-intensity interval exercise reduces human monocyte Toll-like receptor 2 expression in type 2 diabetes. *American journal of physiology. Regulatory, integrative and comparative physiology*, *312*(4), R529–R538. https://doi.org/10.1152/ajpregu.00348.2016	No control group
Eken, Ö., & Kafkas, M. E. (2022). Effects of low and high intensity interval training exercises on VO_{2max} and components of neuromuscular and vascular system in male volunteers. *Journal of musculoskeletal & neuronal interactions*, *22*(3), 352–363.	No eligible intervention
Elliott, B. T., Herbert, P., Sculthorpe, N., Grace, F. M., Stratton, D., & Hayes, L. D. (2017). Lifelong exercise, but not short-term high-intensity interval training, increases GDF11, a marker of successful aging: a preliminary investigation. *Physiological reports*, *5*(13), e13343. https://doi.org/10.14814/phy2.13343	No control group
Enette, L., Vogel, T., Merle, S., Valard-Guiguet, A.-G., Ozier-Lafontaine, N., Neviere, R., Leuly-Joncart, C., Fanon, J. L., & Lang, P. O. (2020). Effect of 9 weeks continuous vs. interval aerobic training on plasma BDNF levels, aerobic fitness, cognitive capacity and quality of life among seniors with mild to moderate Alzheimer's disease: a randomized controlled trial. *European Review of Aging and Physical Activity*, *17*, 1–16.	Combined with medication treatment
Enríquez-Schmidt, J., Mautner Molina, C., Kalazich Rosales, M., Muñoz, M., Ruiz-Uribe, M., Fuentes Leal, F., Monrroy Uarac, M., Cárcamo Ibaceta, C., Fazakerley, D. J., Larance, M., Ehrenfeld, P., & Martínez-Huenchullán, S. (2024). Moderate-intensity constant or high-intensity interval training? Metabolic effects on candidates to undergo bariatric surgery. *Nutrition, Metabolism and Cardiovascular Diseases*. https://doi.org/10.1016/j.numecd.2024.03.001	No eligible intervention
Farinha, J. B., Ramis, T. R., Vieira, A. F., Macedo, R. C. O., Rodrigues-Krause, J., Boeno, F. P., Schroeder, H. T., Müller, C. H., Boff, W., Krause, M., De Bittencourt, P. I. H., Jr, & Reischak-Oliveira, A. (2018). Glycemic, inflammatory and oxidative stress responses to different high-intensity training protocols in type 1 diabetes: A	No eligible comparator

randomized clinical trial. *Journal of diabetes and its complications*, *32*(12), 1124–1132. https://doi.org/10.1016/j.jdiacomp.2018.09.008	
Farley, M. J., Boytar, A. N., Adlard, K. N., Salisbury, C. E., Hart, N. H., Schaumberg, M. A., Jenkins, D. G., & Skinner, T. L. (2024). Interleukin-15 and high-intensity exercise: relationship with inflammation, body composition and fitness in cancer survivors. *The Journal of physiology*, *602*(20), 5203–5215. https://doi.org/10.1113/JP286043	No control group
Fry, R. W., Morton, A. R., & Keast, D. (1992). Acute intensive interval training and T-lymphocyte function. *Medicine and science in sports and exercise*, *24*(3), 339–345.	No control group
Gökçe, E., Adıgüzel, E., Koçak, Ö. K., Kılınç, H., Langeard, A., Boran, E., & Cengiz, B. (2024). Impact of Acute High-intensity Interval Training on Cortical Excitability, M1-related Cognitive Functions, and Myokines: A Randomized Crossover Study. *Neuroscience*, *551*, 290–298. https://doi.org/10.1016/j.neuroscience.2024.05.032	Excluded due to lacking information about some eligibility criteria (i.e. others)
Hajizadeh Maleki, B., Tartibian, B., & Chehrazi, M. (2017). The effects of three different exercise modalities on markers of male reproduction in healthy subjects: a randomized controlled trial. *Reproduction (Cambridge, England)*, *153*(2), 157–174. https://doi.org/10.1530/REP-16-0318	No eligible outcome
Henke E, Oliveira VS, Silva IMd, et al. Acute and chronic effects of High Intensity Interval Training on inflammatory and oxidative stress markers of postmenopausal obese women. *Transl Sports Med*. 2018; 1: 257–264. https://doi.org/10.1002/tsm2.43	No control group
Hooshmand Moghadam, B., Golestani, F., Bagheri, R., Cheraghloo, N., Eskandari, M., Wong, A., Nordvall, M., Suzuki, K., & Pournemati, P. (2021). The Effects of High-Intensity Interval Training vs. Moderate-Intensity Continuous Training on Inflammatory Markers, Body Composition, and Physical Fitness in Overweight/Obese Survivors of Breast Cancer: A Randomized Controlled Clinical Trial. *Cancers*, *13*(17), 4386. https://doi.org/10.3390/cancers13174386	Combined with medication treatment
Hsu, C. C., Fu, T. C., Huang, S. C., & Wang, J. S. (2020). High-intensity interval training recuperates capacity of endogenous thrombin generation in heart failure patients with reduced ejection fraction. *Thrombosis research*, *187*, 159–165. https://doi.org/10.1016/j.thromres.2020.01.013	Excluded due to lacking information about some eligibility criteria (i.e. others)
Hsu, C.-C., Fu, T.-C., Huang, S.-C., Chen, C. P.-C., & Wang, J.-S. (2020). Increased serum brain-derived neurotrophic factor with high-intensity interval training in stroke patients: A randomized controlled trial. *Annals of Physical and Rehabilitation Medicine*, *64*(4), 101385. https://doi.org/10.1016/j.rehab.2020.03.010	Combined with medication treatment
Inoue, D. S., Monteiro, P. A., Gerosa-Neto, J., Santana, P. R., Peres, F. P., Edwards, K. M., & Lira, F. S. (2020). Acute increases in brain-derived neurotrophic factor following high or moderate-intensity exercise is	No eligible outcome

accompanied with better cognition performance in obese adults. Scientific reports, 10(1), 13493. https://doi.org/10.1038/s41598-020-70326-1	
Isanejad, A., Nazari, S., Gharib, B., & Motlagh, A. G. (2023). Comparison of the effects of high-intensity interval and moderate-intensity continuous training on inflammatory markers, cardiorespiratory fitness, and quality of life in breast cancer patients. Journal of sport and health science, 12(6), 674–689. https://doi.org/10.1016/j.jshs.2023.07.001	Combined with medication treatment
Jäger, A., Pieper, A., Priebe, K., Hellweg, R., Meyer, K., Herrmann, S., Wolfarth, B., Grummt, M., Ströhle, A., & Schoofs, N. (2024). Effects of high intensity interval training on serum brain-derived neurotrophic factor in individuals with PTSD. *Journal of psychiatric research*, *180*, 355–361. https://doi.org/10.1016/j.jpsychires.2024.11.009	Combined with medication treatment
Kim, S., Lee, S., Han, D., Jeong, I., Lee, H. H., Koh, Y., Chung, S. G., & Kim, K. (2023). One-year Aerobic Interval Training Improves Endothelial Dysfunction in Patients with Atrial Fibrillation: A Randomized Trial. *Internal medicine (Tokyo, Japan)*, *62*(17), 2465–2474. https://doi.org/10.2169/internalmedicine.0947-22	Combined with medication treatment
Kim, J. S., Taaffe, D. R., Galvão, D. A., Hart, N. H., Gray, E., Ryan, C. J., Kenfield, S. A., Saad, F., & Newton, R. U. (2022). Exercise in advanced prostate cancer elevates myokine levels and suppresses in-vitro cell growth. *Prostate cancer and prostatic diseases*, *25*(1), 86–92. https://doi.org/10.1038/s41391-022-00504-x	No eligible intervention
Hamza Kucuk, Mehmet Soyler, Tulay Ceylan, Levent Ceylan, Fatma Nese Sahin. Effects of acute and chronic high-intensity interval training on serum irisin, BDNF and apelin levels in male soccer referees. Journal of Men's Health. 2024; 20(2): 120-125. doi: 10.22514/jomh.2024.027.	No control group
Kazemi, N., Afrasyabi, S., & Mohamadi Zadeh, M. A. (2024). The effects of high intensity interval training induced H2O2, Nrf2 changes on antioxidants factors in type 2 diabetes. *Journal of Diabetes and Metabolic Disorders*, *23*(2), 1829–1838. https://doi.org/10.1007/s40200-022-01128-7	Combined with medication treatment
Kovacevic, A., Fenesi, B., Paolucci, E., & Heisz, J. J. (2020). The effects of aerobic exercise intensity on memory in older adults. *Applied Physiology, Nutrition and Metabolism*, *45*(6), 591–600. https://doi.org/10.1139/apnm-2019-0495	Combined with medication treatment
Kujach, S., Chroboczek, M., Jaworska, J. *et al.* Judo training program improves brain and muscle function and elevates the peripheral BDNF concentration among the elderly. *Sci Rep* **12**, 13900 (2022). https://doi.org/10.1038/s41598-022-17719-6	No eligible intervention
Kurgan, N., Noaman, N., Pergande, M. R., Cologna, S. M., Coorssen, J. R., & Klentrou, P. (2019). Changes to the Human Serum Proteome in Response to High Intensity Interval Exercise: A Sequential Top-Down Proteomic Analysis. *Frontiers in physiology*, *10*, 362. https://doi.org/10.3389/fphys.2019.00362	No control group

Lee, M.-C., Chung, Y.-C., Thenaka, P. C., Wang, Y.-W., Lin, Y.-L., & Kan, N.-W. (2024). Effects of different HIIT protocols on exercise performance, metabolic adaptation, and fat loss in middle-aged and older adults with overweight. *International Journal of Medical Sciences*, *21*(9), 1689–1700. https://doi.org/10.7150/ijms.96073	Excluded due to lacking information about some eligibility criteria (i.e. others)
Mallard, A. R., Hollekim-Strand, S. M., Coombes, J. S., & Ingul, C. B. (2017). Exercise intensity, redox homeostasis and inflammation in type 2 diabetes mellitus. *Journal of science and medicine in sport*, *20*(10), 893–898. https://doi.org/10.1016/j.jsams.2017.03.014	Excluded due to lacking information about some eligibility criteria (i.e. others)
Martínez-Díaz, I. C., Escobar-Muñoz, M. C., & Carrasco, L. (2020). Acute Effects of High-Intensity Interval Training on Brain-Derived Neurotrophic Factor, Cortisol and Working Memory in Physical Education College Students. *International journal of environmental research and public health*, *17*(21), 8216. https://doi.org/10.3390/ijerph17218216	No control group
Moholdt, T., Aamot, I. L., Granøien, I., Gjerde, L., Myklebust, G., Walderhaug, L., Brattbakk, L., Hole, T., Graven, T., Stølen, T. O., Amundsen, B. H., Mølmen-Hansen, H. E., Støylen, A., Wisløff, U., & Slørdahl, S. A. (2012). Aerobic interval training increases peak oxygen uptake more than usual care exercise training in myocardial infarction patients: a randomized controlled study. *Clinical rehabilitation*, *26*(1), 33–44. https://doi.org/10.1177/0269215511405229	No eligible comparator
Moholdt, T. T., Amundsen, B. H., Rustad, L. A., Wahba, A., Løvø, K. T., Gullikstad, L. R., Bye, A., Skogvoll, E., Wisløff, U., & Slørdahl, S. A. (2009). Aerobic interval training versus continuous moderate exercise after coronary artery bypass surgery: a randomized study of cardiovascular effects and quality of life. *American heart journal*, *158*(6), 1031–1037. https://doi.org/10.1016/j.ahj.2009.10.003	Combined with medication treatment
Mokhtarzade, M., Ranjbar, R., Majdinasab, N., Patel, D., & Molanouri Shamsi, M. (2017). Effect of aerobic interval training on serum IL-10, TNFα, and adipokines levels in women with multiple sclerosis: possible relations with fatigue and quality of life. *Endocrine*, *57*(2), 262–271. https://doi.org/10.1007/s12020-017-1337-y	No eligible intensity
Munk, P. S., Breland, U. M., Aukrust, P., Ueland, T., Kvaløy, J. T., & Larsen, A. I. (2011). High intensity interval training reduces systemic inflammation in post-PCI patients. *European journal of cardiovascular prevention and rehabilitation : official journal of the European Society of Cardiology, Working Groups on Epidemiology & Prevention and Cardiac Rehabilitation and Exercise Physiology*, *18*(6), 850–857. https://doi.org/10.1177/1741826710397600	Combined with medication treatment
O'Neill, C. D., O'Rourke, N., Jeffrey, M., Green-Johnson, J. M., & Dogra, S. (2022). Salivary concentrations of IL-8 and IL-1ra after HIIT and MICT in young, healthy adults: A randomized exercise study. *Cytokine*, *157*, 155965. https://doi.org/10.1016/j.cyto.2022.155965	No eligible outcome

Ramos, J. S., Dalleck, L. C., Stennett, R. C., Mielke, G. I., Keating, S. E., Murray, L., Hasnain, S. Z., Fassett, R. G., McGuckin, M., Croci, I., & Coombes, J. S. (2020). Effect of Different Volumes of Interval Training and Continuous Exercise on Interleukin-22 in Adults with Metabolic Syndrome: A Randomized Trial. *Diabetes, metabolic syndrome and obesity : targets and therapy*, *13*, 2443–2453. https://doi.org/10.2147/DMSO.S251567	Combined with medication treatment
Rezaeimanesh, D. (2022). The effect of a period of high-intensity interval swimming training on plasma levels of myonectin, insulin-like growth factor-1 (IGF-1), and lipid profile in overweight men	Excluded due to lacking information about some eligibility criteria (i.e. others)
Robinson, E., Durrer, C., Simtchouk, S., Jung, M. E., Bourne, J. E., Voth, E., & Little, J. P. (2015). Short-term high-intensity interval and moderate-intensity continuous training reduce leukocyte TLR4 in inactive adults at elevated risk of type 2 diabetes. *Journal of applied physiology (Bethesda, Md. : 1985)*, *119*(5), 508–516. https://doi.org/10.1152/japplphysiol.00334.2015	Combined with medication treatment
Sarkar, S., Debnath, M., Das, M., Bandyopadhyay, A., Dey, S. K., & Datta, G. (2021). Effect of high intensity interval training on antioxidant status, inflammatory response and muscle damage indices in endurance team male players. *Apunts Sports Medicine*, *56*(210). https://doi.org/10.1016/j.apunsm.2021.100352	No eligible intervention
Shing, C. M., Webb, J. J., Driller, M. W., Williams, A. D., & Fell, J. W. (2013). Circulating adiponectin concentration and body composition are altered in response to high-intensity interval training. *The Journal of Strength & Conditioning Research*, *27*(8), 2213–2218.	No eligible intervention
Soltani, N., Marandi, S. M., Kazemi, M., & Esmaeil, N. (2020). Meta-inflammatory state and insulin resistance can improve after 10 weeks of combined all-extremity high-intensity interval training in sedentary overweight/obese females: a quasi-experimental study. *Journal of Diabetes and Metabolic Disorders*, *19*(2), 717–726. https://doi.org/10.1007/s40200-020-00550-z	No eligible intervention
Steckling, F. M., Farinha, J. B., Figueiredo, F. D., dos Santos, D. L., Bresciani, G., Kretzmann, N. A., Stefanello, S. T., Courtes, A. A., Beck, M. D., Cardoso, M. S., Duarte, M., Moresco, R. N., & Soares, F. A. A. (2019). High-intensity interval training improves inflammatory and adipokine profiles in postmenopausal women with metabolic syndrome. *ARCHIVES OF PHYSIOLOGY AND BIOCHEMISTRY*, *125*(1), 85–91. https://doi.org/10.1080/13813455.2018.1437750	No control group
Stensvold, D., Slørdahl, S. A., & Wisløff, U. (2012). Effect of exercise training on inflammation status among people with metabolic syndrome. *Metabolic Syndrome and Related Disorders*, *10*(4), 267–272. https://doi.org/10.1089/met.2011.0140	Combined with medication treatment
Trepci, A., Imbeault, S., Wyckelsma, V. L., Westerblad, H., Hermansson, S., Andersson, D. C., Piehl, F., Venckunas, T., Brazaitis, M., Kamandulis, S., Brundin, L., Erhardt, S., & Schwieler, L. (2020). Quantification	No control group

of Plasma Kynurenine Metabolites Following One Bout of Sprint Interval Exercise. *International Journal of Tryptophan Research*, *13*. https://doi.org/10.1177/1178646920978241	
Windsor, M. T., Bailey, T. G., Perissiou, M., Greaves, K., Jha, P., Leicht, A. S., Russell, F. D., Golledge, J., & Askew, C. D. (2018). Acute Inflammatory Responses to Exercise in Patients with Abdominal Aortic Aneurysm. *Medicine and Science in Sports and Exercise*, *50*(4), 649–658. https://doi.org/10.1249/MSS.0000000000001501	Combined with medication treatment
