# Supplementary material for: Acute and chronic effects of high-intensity interval training on selected exerkine secretion in health, disease, and aging: a systematic review
Source: Front Physiol. 2026 Jan 23;16:1733269. doi: 10.3389/fphys.2025.1733269 (PMC12875996; doi:10.3389/fphys.2025.1733269)
Supplement: Supplementary file 2 [file DataSheet3.pdf]

Table S2. Database search strategy.

**Search set for Medline (via Pudmed)**
(((("High-Intensity Interval Training"[All Fields] OR "HIIT"[All Fields] OR "Interval Training"[All Fields] OR "Sprint Interval Training"[All Fields]) AND ("Exerkines"[All Fields] OR "Myokines"[All Fields] OR "Cytokines"[All Fields] OR "Neuroproteins"[All Fields] OR "muscle-derived cytokines"[All Fields] OR "immune signaling proteins"[All Fields] OR "neuroprotective proteins"[All Fields] OR "BDNF"[All Fields] OR "VEGF"[All Fields] OR "IGF-1"[All Fields] OR "FGF21"[All Fields] OR "adiponectin"[All Fields] OR "myostatin"[All Fields] OR "decorin"[All Fields] OR "interleukins"[All Fields] OR "kynurenine pathway metabolites"[All Fields] OR "tryptophan metabolites"[All Fields] OR "tryptophan catabolites"[All Fields] OR "kynurenine"[All Fields]) AND ("effects"[All Fields] OR "impact"[All Fields] OR "influence"[All Fields]) AND ("humans"[All Fields] OR "adult humans"[All Fields] OR "healthy individuals"[All Fields] OR "athletes"[All Fields] OR "patients"[All Fields])) NOT ("animals"[All Fields] OR "animal studies"[All Fields] OR "rats"[All Fields] OR "mice"[All Fields])) AND "controlled clinical trial"[Publication Type] AND "controlled clinical trial"[Publication Type]) AND (controlledclinicaltrial[Filter])
**Total results: 51**
**Search set for Web of Science**
ALL=(("High-Intensity Interval Training" OR "HIIT" OR "Interval Training" OR "Sprint Interval Training") AND ("exerkines" OR "Myokines" OR "Cytokines" OR "neuroproteins" OR "muscle-derived cytokines" OR "immune signaling proteins" OR "neuroprotective proteins" OR "BDNF" OR "VEGF" OR "IGF-1" OR "FGF21" OR "adiponectin" OR "myostatin" OR "decorin" OR "interleukins" OR "kynurenine pathway metabolites" OR "tryptophan metabolites" OR "tryptophan catabolites" OR "kynurenine") AND ("effects" OR "impact" OR "influence") AND ("humans" OR "adult humans" OR "healthy individuals" OR "athletes" OR "patients") NOT ("animals" OR "animal studies" OR "rats" OR "mice"))
**Total results**: 40
**Search set for Google Scholar**
allintitle: "High-Intensity Interval Training" OR "HIIT" OR "Interval Training" OR "Sprint Interval Training" AND "Exerkines" OR "Myokines" OR "Cytokines" OR "Neuroproteins" OR "muscle-derived cytokines" OR "immune signaling proteins" OR "neuroprotective proteins" OR "BDNF" OR "VEGF" OR "IGF-1" OR "FGF21" OR "adiponectin" OR "myostatin" OR "decorin" OR "interleukins" OR "kynurenine pathway metabolites" OR "tryptophan metabolites" OR "tryptophan catabolites" OR "kynurenine" AND "effects" OR "impact" OR "influence" AND "humans" OR "adult humans" OR "healthy individuals" OR "athletes" OR "patients"
**Total results:** 150
**Search set for Scopus**

TITLE-ABS-KEY ("High-Intensity Interval Training" OR "hit" OR "Interval Training" OR "Sprint Interval Training") AND TITLE-ABS-KEY ("exerkines" OR "cytokines" OR "ferroproteins" OR "myokines" OR "immune signaling proteins" OR "neuroprotective proteins" OR "BDNF" OR "VEGF" OR "IGF-1" OR "FGF-21" OR "adiponectin" OR "myostatin" OR "decorin" OR "interleukins" OR "kynurenine pathway metabolites" OR "tryptophan metabolites" OR "kynurenine") AND TITLE-ABS-KEY ("effects" OR "impact" OR "influence") AND TITLE-ABS-KEY ("humans" OR "adult humans" OR "healthy individuals" OR "athletes" OR "patients") AND NOT TITLE-ABS-KEY ("animals" OR "animal studies" OR "rats" OR "mice") AND (LIMIT-TO (DOCTYPE , "ar")) AND (LIMIT-TO (EXACTKEYWORD , "Human"))
**Total results**: 610
