# Supplementary material for: Acute and chronic effects of high-intensity interval training on selected exerkine secretion in health, disease, and aging: a systematic review
Source: Front Physiol. 2026 Jan 23;16:1733269. doi: 10.3389/fphys.2025.1733269 (PMC12875996; doi:10.3389/fphys.2025.1733269)
Supplement: Supplementary file 3 [file DataSheet1.pdf]

Risk of bias domains

Study	D1	D2	D3	D4	D5	Overall
Alizadeh 2019	-	-	+	+	+	-
Allen 2017	+	-	+	+	+	-
Banitalebi 2017	+	+	+	+	-	-
Barry 2018	-	-	-	+	+	-
Boyne 2019	-	-	+	+	+	-
Boyne 2020	-	-	+	+	+	-
Coletta 2021	+	+	+	+	+	+
Cooper 2016	-	+	+	+	+	-
De Lima 2022	+	-	+	+	+	-
Elmer 2016	-	+	+	-	-	-
Farrow 2024	+	-	+	+	-	-
Gao 2023	+	-	+	+	+	-
Gerosa-Neto 2016	-	+	+	+	-	-
Haghighi 2022	+	+	-	+	+	-
Heiston 2020	-	+	+	+	-	-
Hoekstra 2017	-	+	+	+	-	-
Hovanloo 2013	-	+	+	+	-	-
Hovsepian 2021	-	+	-	+	-	-
Javelle 2021	+	+	+	+	-	-
Kang 2024	-	+	+	+	+	-
Kaspar 2016	-	+	-	+	-	-
Kordi 2013	-	+	-	-	X	X
Kujach 2020	-	+	+	+	-	-
Lucibello 2020	-	+	+	+	-	-
Marquez 2015	-	+	+	+	-	-
Middelbeek 2021	-	+	+	+	+	-
Peake 2014	-	+	+	+	+	-
Proschinger 2023	+	+	+	+	+	+
Renteria 2019	+	+	+	+	X	X
Richards 2010	-	+	+	+	+	-
Rioux 2024	+	+	+	+	-	-
Rodriguez 2018	-	+	+	+	+	-
Rohnejad 2023	+	-	+	+	+	-
Sasimontonkul 2024	-	+	+	+	-	-
Slusher 2018	-	+	+	+	+	-
Tsai 2021	-	+	+	+	-	-
Wahl 2014	-	+	+	+	-	-
Yang 2024	-	-	+	+	+	-
Zhang 2024	-	+	+	+	+	-

Domains:
D1: Bias arising from the randomization process.
D2: Bias due to deviations from intended intervention.
D3: Bias due to missing outcome data.
D4: Bias in measurement of the outcome.
D5: Bias in selection of the reported result.

Judgement
X High
- Some concerns
+ Low

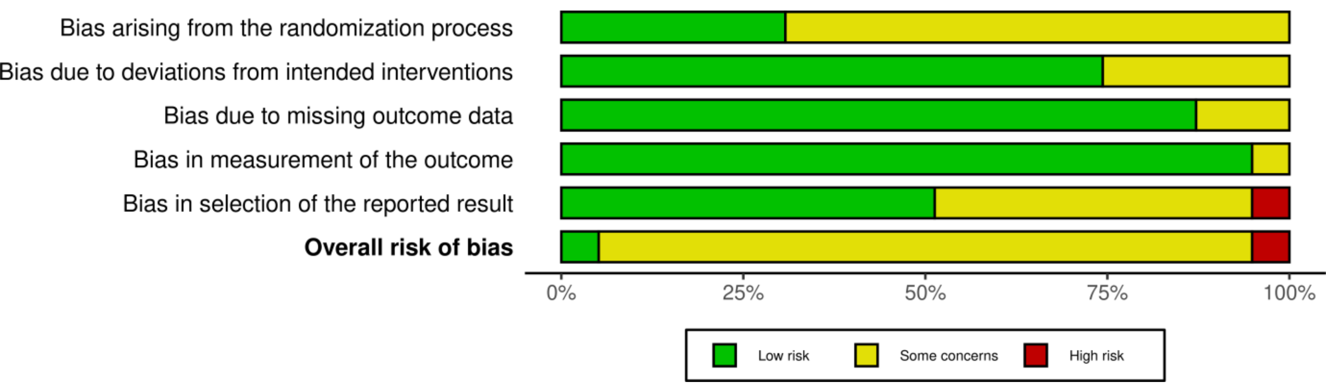

Figure S1. Risk of bias assessment
